# Mitochondrial enzyme FAHD1 reduces ROS in osteosarcoma

**DOI:** 10.1038/s41598-024-60012-x

**Published:** 2024-04-22

**Authors:** Anne Heberle, Elia Cappuccio, Andreas Andric, Tatjana Kuen, Anna Simonini, Alexander K. H. Weiss

**Affiliations:** Institute for Biomedical Aging Research, University of Innsbruck, Innsbruck, Austria

**Keywords:** Enzymes, Bone cancer

## Abstract

This study investigated the impact of overexpressing the mitochondrial enzyme *Fumarylacetoacetate hydrolase domain-containing protein 1* (FAHD1) in *human osteosarcoma epithelial cells* (U2OS) in vitro. While the downregulation or knockdown of FAHD1 has been extensively researched in various cell types, this study aimed to pioneer the exploration of how increased catalytic activity of *human FAHD1 isoform 1* (hFAHD1.1) affects human cell metabolism. Our hypothesis posited that elevation in FAHD1 activity would lead to depletion of mitochondrial oxaloacetate levels. This depletion could potentially result in a decrease in the flux of the tricarboxylic acid (TCA) cycle, thereby accompanied by reduced ROS production. In addition to hFAHD1.1 overexpression, stable U2OS cell lines were established overexpressing a catalytically enhanced variant (T192S) and a loss-of-function variant (K123A) of hFAHD1. It is noteworthy that homologs of the T192S variant are present in animals exhibiting increased resistance to oxidative stress and cancer. Our findings demonstrate that heightened activity of the mitochondrial enzyme FAHD1 decreases cellular ROS levels in U2OS cells. However, these results also prompt a series of intriguing questions regarding the potential role of FAHD1 in mitochondrial metabolism and cellular development.

## Introduction

### The multifaceted role of ROS in osteosarcoma pathogenesis

*Reactive oxygen species* (ROS) play a multifaceted role in the pathogenesis of osteosarcoma, a primary malignant bone tumour^1,2^. ROS serve as signalling molecules in the osteosarcoma microenvironment, modulating cellular processes such as proliferation, survival, angiogenesis, and metastasis. Dysregulated ROS production promotes tumour cell proliferation by activating growth-promoting pathways while inhibiting apoptosis through redox-sensitive mechanisms^2^. Elevated ROS levels are implicated in oncogenic transformation, contributing to genetic instability and the accumulation of mutations, hallmark features of cancer development. ROS induce DNA damage, promoting the activation of oncogenes and the inactivation of tumour suppressor genes, crucial events in the initiation and progression of osteosarcoma^2^.

ROS play a paradoxical role in the therapy response of osteosarcoma^1,2^. While ROS induction is utilized in chemotherapy and radiotherapy to induce cytotoxicity, high ROS levels may also confer resistance to therapy through the activation of antioxidant defence mechanisms in cancer cells. This intricate balance between ROS-mediated cytotoxicity and adaptation underscores the complexity of targeting ROS in osteosarcoma treatment^1^. Understanding the critical link between ROS and osteosarcoma offers potential therapeutic avenues^2^. Targeting ROS production or enhancing ROS-mediated cytotoxicity could be exploited to develop novel anti-osteosarcoma strategies. However, the therapeutic exploitation of ROS must be carefully balanced to avoid exacerbating oxidative stress-related damage to normal tissues. Thus, elucidating the precise role of ROS in osteosarcoma pathogenesis and therapy response remains imperative for developing effective therapeutic interventions against this aggressive bone malignancy^2^.

Agents that reduce ROS in osteosarcoma hold promise as potential therapeutic interventions due to their ability to counteract oxidative stress-induced damage and inhibit tumour progression. These agents function through various mechanisms to restore redox balance and mitigate ROS-mediated oncogenic signalling pathways. Firstly, antioxidants such as vitamin C, vitamin E, and glutathione can directly scavenge ROS, thereby reducing oxidative stress and preventing DNA damage and genomic instability in osteosarcoma cells^3,4^. By neutralizing excess ROS, antioxidants mitigate the activation of redox-sensitive signalling pathways that promote tumour growth and metastasis. Additionally, inhibitors of ROS-generating enzymes, such as NADPH oxidases (NOX) and xanthine oxidase, offer another approach to reduce ROS levels in osteosarcoma^5^. Pharmacological agents targeting these enzymes can attenuate ROS production, thereby inhibiting ROS-mediated oncogenic signalling cascades and suppressing tumour progression. Moreover, compounds that enhance the activity of endogenous antioxidant enzymes, such as superoxide dismutase (SOD) and catalase, represent another strategy to reduce ROS in osteosarcoma^6,7^. By augmenting the cellular antioxidant defence system, these agents bolster the capacity of osteosarcoma cells to detoxify ROS, thereby conferring protection against oxidative stress-induced damage and inhibiting tumour growth. Furthermore, targeting redox-sensitive signalling pathways implicated in osteosarcoma pathogenesis represents a promising therapeutic approach to reduce ROS levels. Inhibitors of signalling molecules such as nuclear factor erythroid 2-related factor 2 (NRF2) and nuclear factor-kappa B (NF-κB) can suppress ROS-induced gene expression and mitigate the pro-tumorigenic effects of oxidative stress in osteosarcoma^8–10^.

Potential therapeutic benefits of agents targeting ROS in osteosarcoma lie in their ability to mitigate oxidative stress-induced damage, inhibit oncogenic signalling pathways, and suppress tumour cell progression. Further research into the efficacy and safety of these agents is crucial for their development as novel treatments for osteosarcoma^1^.

### *Fumarylacetoacetate hydrolase domain-containing protein 1* (FAHD1)

The eukaryotic protein *Fumarylacetoacetate hydrolase domain-containing protein 1* (FAHD1) functions as mitochondrial oxaloacetate decarboxylase^11^ (ODx) (and oxalacetate keto-enol isomerase^12^), playing a crucial role in regulating the flux of the tricarboxylic acid cycle^11,13–15^. FAHD1 belongs to the fumarylacetoacetate hydrolase (FAH) superfamily^13,16,17^. Among the three known FAH family members in eukaryotes that exhibit ODx activity (catalyzing the decarboxylation of oxaloacetate to pyruvate and CO_2_)^11,13^, FAHD1 and FAHD2 (a and b isoforms) are prominent^15^.

In eukaryotes, FAHD enzymes are primarily localized in mitochondria^11^, suggesting their potential antagonistic interaction with *pyruvate carboxylase* (PC)^18^. Furthermore, FAHD enzymes may compete for the oxaloacetate substrate with other enzymes involved in oxaloacetate consumption, such as *citrate synthase* (CS), *malate dehydrogenase* (MDH), and *mitochondrial phosphoenolpyruvate carboxykinase* (PEPCK)^19^. This competition contributes to the regulation of oxaloacetate, a critical metabolite^20,21^.

Prior research has underscored the significant role of the enzyme FAHD1 in the metabolism of various cell types, tissues, and organisms^22–25^. FAHD1 exhibits highest expression levels in the kidney and liver^26^, and its expression appears to correlate with tissue dependence on calcium^15^. Acting as a major regulator of mitochondrial oxaloacetate levels, FAHD1 is anticipated to deplete mitochondrial oxaloacetate under conditions of heightened activity. It is hypothesized that increased FAHD1 activity may lead to reduced tricarboxylic acid cycle flux metabolism, resulting in decreased oxidative ATP production and a general decline in ROS levels.

### FAHD1 overexpression in human osteosarcoma cells

Upregulating the catalytic activity of FAHD1 may induce a phenotype similar to the downregulation of FAHD1, as maintaining constant levels of oxaloacetate is crucial for sustaining a robust and steady flux through the tricarboxylic acid cycle^14^. To explore the impact of FAHD1 overexpression on cell fate, a cell line with low endogenous FAHD1 levels was chosen for investigation, using the *human osteosarcoma cell line* (U2OS) due to its suitability for DNA transfection^27^. In addition to overexpressing *human FAHD1 isoform 1* (hFAHD1.1) in vitro, two catalytically altered variants of the enzyme, hFAHD1.1-T192S and hFAHD1.1-K123A, were introduced as described below. Osteosarcoma cells represent primary malignant tumor cells that primarily metastasize to the lungs^28,29^. They originate directly from osteoid tissue and bone-related mesenchymal cells, exhibiting uncontrolled proliferation^30^. Normally, cells metabolize glucose through glycolysis, with glycolytic products subsequently oxidized via oxidative phosphorylation (OXPHOS) in mitochondria to generate ATP. However, cancer cells typically uncouple these processes, favoring aerobic glycolysis (the Warburg effect)^30,31^. This metabolic shift involves increased glucose uptake, leading to elevated lactate production^32^ even in the presence of oxygen^30^. Aerobic glycolysis is also utilized for generating metabolic intermediates and antioxidants^31,33^, enabling cancer cells to adapt to environmental challenges such as acidification or hypoxia^30^. Additionally, osteosarcoma cells may utilize other nutrients, such as amino acids, to fuel the tricarboxylic acid cycle, serving as biosynthetic precursors^34^.

Feeding amino acids into the tricarboxylic acid cycle allows cancer cells to sustain ATP production via OXPHOS, even when uncoupled^31^. Glutamine, in particular, serves as a critical nitrogen and carbon source for various biosynthetic processes^34^. Glutamine is transported into mitochondria via the ASCT2 (SLC1A5) transporter and converted to glutamate by *glutaminase* (GLS1). Glutamate is then further metabolized into 2-oxoglutarate (α-ketoglutarate), an intermediate of the tricarboxylic acid cycle, along with ammonia, by mitochondrial *glutamate dehydrogenase 1* (GLUD1)^35,36^. 2-oxoglutarate is subsequently oxidized in the tricarboxylic acid cycle to succinate and fumarate, yielding ATP, NADH, and FADH_2_^36^. Moreover, glutamine serves as a nitrogen source for the biosynthesis of important molecules such as alanine, aspartate, serine, or asparagine^34,36,37^. The catalytic activity of asparagine synthetase (ASNS) appears particularly significant, as it is implicated in metastasis and tumorigenesis, correlating with poor survival in various cancer types, including sarcomas^36–38^.

### The FAHD1 variant T192S

A small subset of enzymes within the FAH superfamily exhibit ODx activity^16,17^. The significance of a human FAHD1 T192S variant was identified through a BLAST search against the highly active ODx enzyme Cg1458 from *Corynebacterium glutamicum*^39–41^. Although FAHD1 demonstrates unexpectedly low catalytic activity^13^, Cg1458 exhibits remarkably high catalytic efficiency^40,41^. Analysis revealed that while the catalytic regions of Cg1458 and FAHD1 share conservation, they differ at a single position, where S258 of Cg1458 corresponds to T192 in human FAHD1 (see Fig. S1). The T192S mutation was introduced into human FAHD1, and the ODx activity of the recombinant protein was investigated in vitro, demonstrating enhanced catalytic activity compared to the wild-type protein (see Fig. S2 and S3).

Of significant interest, a surprising correlation was noted between the expression of the T192S variant of FAHD1 in certain animals exhibiting long lifespans and resistance to cancer. This correlation is attributed to a heightened resistance to the detrimental effects of senescence and robust responses to environmental stressors (see Fig. S4). A noteworthy trend towards increased lifespan-to-weight ratio is evident among animals harboring the T192S variant. Specifically, the presence of the T192S variant of FAHD1 is observed in several long-lived bird species. The constraint on lifespan observed in replicative senescence is closely linked to oxidative stress, which likely acts as both a cause and a consequence of impaired mitochondrial respiratory function^42^. The mechanism of cellular senescence entails the permanent arrest of cell division while cellular metabolism persists. It's worth mentioning that, alongside their extended lifespans, all these animals are either uricotelic species or possess enhanced ammonia detoxification capabilities^43^. Birds, for instance, exhibit lower rates of free radical production and oxidative damage compared to mammals, despite their higher levels of oxidative metabolism. This resilience is attributed in part to the abundant presence of the antioxidant uric acid in birds, which serves to mitigate oxidative damage^44^. Turtles are renowned for their longevity, with the phenomenon of negligible senescence observed in certain species. This characteristic involves heightened regenerative capacities in response to environmental stressors, contributing to their remarkable longevity^45^.

### The FAHD1 variant K123A

The enzymatic activity of FAHD1 relies on its inherent oxaloacetate isomerase activity^12^, which is crucial for facilitating both acetylpyruvate hydrolase and ODx functions. In our proposed model, the side chains of K47 and K123 play a vital role in stabilizing the carboxylate side chain of D102^13^. Specifically, the substitution of a lysine residue with alanine (K123A) resulted in the loss of both acetylpyruvate hydrolase and ODx activity, providing strong evidence for the essential role of K123 in catalytic activity^13^. During the decarboxylation process, the primary products of C3-C4 bond cleavage are enol pyruvate and carbon dioxide. The nucleophilic attack of hydroxyl to the electrophilic C4 of the acetyl group must be followed by K123-assisted formation of enol pyruvate, involving decarboxylation and protonation of the Mg^2+^-bound pyruvate enolate by K123. In simpler terms, our model suggests that the resonance-stabilized Mg^2+^ pyruvate-enolate complex generated during the reaction must be converted to the enol form by K123. Failure to do so, such as with the introduction of a K123A mutation, would result in the accumulation of an unquenched pyruvate-enolate complex, ultimately leading to the destabilization of the cavity. This phenomenon is consistent with similar reaction mechanisms observed in related enzymes^46^. a catalytically inactive mutant was observed *in vitro*^13^.

### The PEP-PYR-OAA node may also involve FAHD1 activity

Connecting glycolysis with the tricarboxylic acid *cycle*, the metabolites phosphoenolpyruvate (PEP), pyruvate (PYR), and oxaloacetate (OAA) form the core of a network involving several key enzymes regulating tricarboxylic acid flux and adjacent metabolic pathways^47,48^. The PEP-PYR-OAA node defines a regulatory sub-cycle involving enzymes such as PEPCK-M and PC in the mitochondrial matrix, as well as *pyruvate kinase* (PK) in the cytosol, along with transporters for PEP and PYR. Regulation of this node is critical for cellular energetics, with enzymes involved in (de)phosphorylation of nucleotide phosphates and redox enzymes responsible for malate conversion^47,49^. Additionally, this cycle facilitates the generation of mitochondrial GDP (mtGDP) from mitochondrial GTP (mtGTP), which fuels the *succinate dehydrogenase* (SDH) reaction, converting mtGDP back to mtGTP. This sub-cycle establishes an implicit connection between tricarboxylic acid flux and the PEP-PYR-OAA node^47,50^. It is proposed that FAHD1 activity as ODx contributes to the regulation of this node, potentially acting antagonistically to the PC reaction. Specifically, the hypothesis is that increased levels of oxaloacetate (resulting from FAHD1 depletion) may directly inhibit SDH activity^22,23^, decreased levels of oxaloacetate (resulting from increased FAHD1 activity) may disrupt the PEP-PYR-OAA cycle, consequently reducing mtGDP levels and indirectly inhibiting SDH activity^23^. Decreased levels of oxaloacetate may be restored in the cytosol through various pathways, such as lactate fermentation or pathways generating malate as a precursor metabolite^51^.

### Targeting ROS in Osteosarcoma: Insights from FAHD1 Overexpression

The impact of overexpressing the mitochondrial enzyme FAHD1 in the osteosarcoma cell model U2OS was investigated, resulting in reduced ROS levels. The findings elucidate that FAHD1 regulates the PEP-PYR-OAA node, a pathway identified as highly relevant for osteosarcoma cell progression. Additionally, the study demonstrates that the catalytically enhanced FAHD1 variant T192S, observed in species with longevity and increased cancer resistance, may shift cellular metabolism from glycolysis towards glutaminolysis. These findings suggest that FAHD1 contributes to the regulation of glycolysis and glutaminolysis in these cells, highlighting its potential as a therapeutic target in osteosarcoma treatment. Notably, in all cases of overexpression, cells react to these changes by increasing mitochondrial copy numbers. However, the discussion of these data also addresses the inherent limitations of our study and acknowledges potential avenues for improvement or future exploration and investigation. Furthermore, the relevance of our findings in the context of osteosarcoma treatment is underscored, emphasizing their potential implications for therapeutic development.

## Materials and methods

### Cells used in this study and cell culture

Human osteosarcoma cells (U2OS) (https://www.cellosaurus.org/CVCL_0042) were originally obtained from the American Tissue Culture Collection (*ATCC*® *HTB-96*™) and were maintained at the Heinrich Pette Institute for several years^52^. The cells were used in previous studies. U2OS cells were cultured in full growth medium composed of DMEM low glucose (SIGMA, D5546), supplemented with 10% FCS (REF), 1% penicillin/streptomycin (SIGMA, P4333), 100 µM pyruvate (SIGMA, S8636), 4 mM L-glutamine solution (SIGMA, G7513-100ML), and 1 g/L D-( +)-glucose solution (SIGMA, G8769). The cells were cultured in T75 flasks (Greiner, 658,175) at 37 °C with 5% CO_2_. During the cultivation process, the growth medium was refreshed every other day, and cells were reseeded once they reached approximately 80% confluence. For experiments involving reduced glucose or glutamine levels, glutamine-only or glucose-only medium conditions were prepared accordingly. Additionally, experiments were conducted using either default or dialyzed FCS (*Thermo-Fischer*, A3382001).

### Stable cell line generation via lipofection and blasticidin selection

The *PiggyBac* transposon system obtained from *VectorBuilder* (https://en.vectorbuilder.com) for mammalian gene expression was employed to establish stable overexpression of human FAHD1 isoform 1, as well as its T192S and K123A variants. These vectors were designed to include a blasticidin resistance gene for selection and employed the truncated human FAHD1 promoter region [*5’-tcagcgtccacctcgcaccgtccgccaaccaatcaacgcgctcggctcaacttgttttctccgcgagccccgactgccacgggccgggagacgggcctgggagcggcggcggcggcggcccgagagctgcgccgggagagctgtgccgcgagcctccgcctcttttttggcaccgcacagtggatccctggaggccgagcgccacgccgcccgggtacccggcagatcgcgaggtggaacgggccgtcgctgcgggggacagcgttccgaggcagttggtcctctccaggatgccgtaggcatcagctgaccggcccagcccacgtgactacaggggcactg-a-3’*] to drive the expression of human FAHD1 isoform 1.1 (NP_001018114.1, Q6P587-1), as well as its T192S and K123A variants. Vector maps were provided as part of the supplementary material.

U2OS cells were seeded into six-well tissue culture plates (*FALCON*, 353,046) at a cell density of 2 × 10^5^ cells per well to achieve 50–80% confluence before transfection. On the day of transfection, individual mixtures were prepared, each containing 85 µL of growth medium, 2.5 µg of the PB Transposon Vector, 0.5 µg of the PB transposase RNA, and 2 µL of lipofectamine® 2000 (*Invitrogen*, 11,668–019). Each mixture was incubated for 5 min at room temperature and then incubated at room temperature for an additional 20 min. The growth medium was removed from the cells, and they were washed with PBS. Next, 2 mL of fresh growth medium was applied to each well. After 20 min, the combined solution of the transfection mixture was added to the cells and mixed by gently shaking the plate. To ensure optimal transfection conditions with the PB Transposon System, cells were then incubated at 37 °C for 18 to 24 h. After 24 h, the medium was removed, and the cells were washed again with PBS. Fresh growth medium (2 mL) was applied for another 24 h. 48 h post lipofection, cells were subjected to selection using blasticidin (10 mg/mL; *SIGMA* 15,205). After approximately a week of selection, all control cells died, while stable cell lines proliferated.

### Growth curve experiments and colony forming assay

To assess the proliferation behavior of the different cell lines, the following experiments were conducted: Individual cell lines were cultured in three different culture media, both with default and dialyzed FCS (*SIGMA*, F0392). The cells were cultured in complete, glutamine-only, and glucose-only medium in 6-well tissue culture plates (*FALCON*, 353,046) for a duration of 16 days. The complete medium was prepared as described in §2.1. Every fourth day, cell counts were performed using a Bio-Rad TC20 cell counter, and the cells were re-seeded with a cell density of 5 × 10^4^ cells per well. Total cell numbers were recorded at each counting day, and the *cumulative population doubling levels* (cPDL) were calculated. Additionally, colony-forming assays were performed using U2OS cells following a previously described protocol^53^. Cells were cultured in triplicates in 6-well tissue culture plates (*FALCON*, 353,046), with 100 cells seeded per well on day 0. The cells were cultured for 15 days with medium changes every four days. On the 15th day, the medium was removed, and cells were washed with PBS (*SIGMA*, D8537-500ML). Formed colonies were fixed in methanol for 5 min at room temperature. After fixation, cells were washed with PBS and stained with 0.5% crystal violet solution for 20 min at room temperature. Excess staining solution was removed by washing the cells multiple times with ddH_2_O. The wells were left to dry, and the area covered by colonies, as well as the intensity of the colonies, were determined using the *ColonyArea* plugin in the *ImageJ* software^53^.

### Preparations of cell lysates, SDS-PAGE and Western blot analysis

For the preparation of cell lysates, at least 10^6^ cells were seeded in a 10 cm petri dish (*Greiner*, 633,181) overnight. The medium was removed, and the cells were washed with PBS. The dish was placed on ice, and 100 µL RIPA buffer were added (e.g., see https://www.novusbio.com/support/general-support/buffers.html). Cells were scratched for about 5 min from the dish using a cell lifter (*costar*®, 3008). The supernatant was collected and transferred into a 1.5 mL tube. In an alternative approach, cells were collected, centrifuged for 5 min at 250 × g, and the medium removed. Cell pellets were then resuspended in 100 µL RIPA buffer (e.g., https://www.novusbio.com/support/general-support/buffers.html). Either way, cells were eventually lysed by friction force (sonication) and centrifuged to remove any cell debris. The supernatant was collected and transferred to a new 1.5 mL tube, while the tube containing the pellet was disposed of. Protein concentration was determined using a BCA Assay. Protein content in any sample was determined using the *Pierce™* BCA protein assay kit (*Thermo Fischer*, 23,227). The standard was prepared using a BSA stock (2 mg/mL) in distilled water. According to the vendor's SOP, 100 µL of reaction solution was used together with 5 µL of sample or standard in a 96-well setup. For SDS-page samples, 30 µg per sample were prepared and mixed with 4 × SDS sample buffer up to a final volume of 20–30 µL. 12.5% SDS-gels were used to efficiently separate the bands of proteins. Casted gels were either used immediately or stored at 4 °C for up to a week wrapped in wet paper. Proteins were separated via SDS-PAGE for at least 2 h at 120 V until the 25 kDa band reached the edge of the gel. Western blot transfer was performed via electroblotting onto activated PVDF membranes (*Bio-Rad*, 16,201,777). The transfer was conducted at a constant of 0.3 A for one hour. To block unspecific binding sites of proteins, membranes were incubated for at least an hour in blocking milk (5% skim milk in PBS with 1% Tween-20). Membranes were then cut according to the sizes of proteins. Primary antibodies were applied to the membrane by transferring the pieces to 50 mL tubes (*FALCON*, 352,070) filled with antibody solutions in blocking milk. In particular, Western blot analysis was performed for pyruvate carboxylase (PC, Proteintech® 16,588–1-AP, 1:1000), mitochondrial phosphoenolpyruvate carboxykinase (PEPCK-M, Santa Cruz sc-32879, 1:2000), glutaminase (GLS) isoforms kidney-type glutaminase (KGA) and glutaminase C (GAC) (Proteintech® 12,855–1-AP, 1:5000), β-actin (SIGMA #A5441, 1:20,000), lactate dehydrogenase B (LDHB, BioLegend® 859,801, 1:5000), and FAHD1 (in-house α-hFAHD1 antibody, 1:500). Antibodies were then incubated at 4 °C overnight. On the following day, primary antibodies were removed, and the membranes were washed for an hour with PBS-T (PBS with 1% Tween-20), changing the washing solution every ten minutes. Secondary antibodies (*Dako*, P0399, P0447) were applied for 45 min at room temperature, followed by another series of 10-min washing steps with PBS-T for about an hour. Finally, membranes were washed with PBS for 5 min to remove residues of Tween-20. After washing, the development solution ECL (*Amersham*, RPN2232) was added, and the membrane was developed at different exposure times via X-ray film.

### FACS experiments to explore ROS levels

To investigate changes in ROS levels, cells were stained with dihydroethidium (hydroethidine) (DHE) (*Invitrogen*, D23107), and ROS levels were measured via flow cytometry analysis. Cells were collected in DMEM without phenol red (*GIBCO*, A14430-01), as phenol red can cause problems in FACS experiments, supplemented as described in §2.1. Cells were counted and aliquoted in triplicates with 3 × 10^5^ cells per FACS tube (*FALCON*, 352,052), while two additional flasks served as positive and unstained negative controls. After distributing the cells into FACS tubes, they were pelleted via centrifugation at 300 × g for 5 min. Meanwhile, fresh medium was prepared containing all supplements except FCS, as FCS can interfere with FACS analysis. Following centrifugation, the supernatant was carefully discarded into the sink without disturbing the cell pellet at the bottom of the flasks. To remove any remaining trypsin and FCS, cells were washed with 1 mL of PBS and carefully suspended to avoid stressing the cells. Cells were resuspended and pelleted again via centrifugation at 300 × g for 5 min. Staining solutions with an FCS-free medium were prepared in the dark (20 µM of DHE). After centrifugation, the supernatant was discarded, and cells were resuspended in 1 mL of staining solution. One aliquot of the control cells was treated with the rotenone-containing staining solution (500 nM, positive control), while another was suspended with FCS- and DHE-free medium (negative control). Cells were covered in aluminum foil and incubated for 30 min at 37 °C. Following incubation, settled cells were resuspended and centrifuged again at 300 × g for 5 min. The supernatant was discarded, and cells were resuspended in 1 mL PBS to remove residues of the staining solution. After centrifugation at 300 × g for 5 min, PBS was discarded, and cells were resuspended in 500 µL PBS for FACS analysis (*BD FACS Canto II*). The DHE-positive cells were measured using the PE channel. After the experiment, the mean DHE intensity of cells was determined using the *FlowJo* software analysis and normalized to mitochondrial content, which was established via qPCR.

An expert recommendation^54^ emphasizes the use of DHE or MitoSOX probes with validated specific products, minimizing probe concentration, and incorporating controls for membrane potentials. LC–MS methods are suggested for precise measurement of all modified species. It is worth noting that fluorescence staining, and quantification were conducted via FACS experiments rather than imaging. DHE is a commonly used agent in Flow Cytometry for ROS detection. Despite its challenges, reliable quantification is ensured through stringent controls. Background fluorescence is gauged by negative controls, while positive controls validate assay sensitivity to ROS. Gating strategies are employed to exclude debris and non-viable cells, ensuring accurate quantification. Although challenges such as fluorescence overlap and non-specific oxidation products are presented by DHE, reliable ROS quantification is facilitated by meticulous experimental design with appropriate controls. Regarding MitoSOX, caution is exercised due to its limitations. Challenges such as potential fluorescence misinterpretation caused by both specific and non-specific oxidation products with overlapping spectra, including ethidium and 2-hydroxyethidium, are posed by the method. Additionally, factors such as membrane potentials and mitochondrial characteristics can influence probe accumulation and fluorescence quenching, potentially affecting measurement reliability. Moreover, fluorescence amplification and artifact creation may occur due to MitoSOX oxidation products intercalating into DNA.

### Immunofluorescence staining and live cell imaging

About 10^5^ cells were seeded on 20 mm coverslips (*Epredia*, CB00200RA120MNZ0) in a 6-well plate (*FALCON*, 353,046), containing 2 mL of growth medium. After the attachment of cells on the coverslips, the medium was removed, and the cells were washed two times with warm PBS. The coverslips were fixated for 20 min at room temperature using 4% ice-cold paraformaldehyde (PFA) solution. After fixation, PFA was removed by four washing steps with cold PBS using a 1 mL pipette. After this step, coverslips were processed immediately or kept at 4 °C in PBS sealed in parafilm for one week. For processing cells, cells were permeabilized in permeabilization solution (PBS containing 0.3% Triton-X and 0.1% Sodium Citrate) by shaking for 3 min at room temperature. Unspecific binding sites were blocked for 20 min at room temperature in 2% BSA dissolved in PBS.

During the 20 min time gap, specimen slides were prepared by labeling and wrapping them in parafilm, as well as primary antibody solutions in blocking solution. Staining was performed with Complex V (red, Invitrogen A2135, 1:250) and FAHD1 (green, in-house α-hFAHD1 antibody, 1:250) antibodies. After blocking, 50 µL of the primary antibody was pipetted on the slide. Coverslips were put on top of the solution, with cells facing the solution, respectively. Slides were incubated at 4 °C overnight or for 1 h at room temperature in a moisturized chamber. After incubation cells were washed four times in PBS and the primary antibody solution was removed from the slide for possible reuse. Cells were incubated in a secondary antibody solution, prepared in blocking solution (1:500; Alexa fluor α-rabbit 488 and 546; *Invitrogen* A11008 and A11003). Incubation was performed for one hour at room temperature in the moisturized chamber. Afterward, coverslips were transferred back to the 6-well plate and washed four times with PBS. Nuclei were counter-stained using DAPI (1:1000 in PBS, Invitrogen 10,184,322, 1:1000) for 5 min in the moisturized chamber. After the staining, cells were moved back to the 6-well plate and washed four times using PBS and two times with ddH_2_O. Parafilm was removed from the slide, the slide was cleaned and 10 µL of fluorescence mounting media (*Dako*, S302380-2) was added. The coverslip (cells facing the mounting media) was put on top and let dry at room temperature in the dark overnight or for at least 5 h. Images were taken using a confocal live cell microscope (*Imsol*, CV1000) and corrected total cell fluorescence (CTCF) was calculated using ImageJ and Excel.

### Glycolytic stress test with the Agilent Seahorse analyzer

Experiments were performed according to the specification in the Agilent Seahorse XF Glycolysis Stress Test Kit (https://www.agilent.com). A day prior to an experiment, the cells were seeded with a cell number of 1 × 10^4^ cells/well in an XFp Cell Culture Miniplate, in technical triplicates. The XFp Sensor Cartridges were prepared with cell culture grade water (*SIGMA*, W3500), and left overnight at 37 °C without CO_2_, to rehydrate the sensor cartridges. On the day of the experiment, cartridges were taken from the incubator, and the water was exchanged with calibrant solution (*Agilent Technologies*, 103,059–000) and put back in the CO_2_-free incubator, one hour prior to the experiment. After 30 min the culture miniplate, containing the cells, was taken and the full medium exchanged by XF base medium (*Agilent Technologies*, 103,575–100) and incubated together with the cartridge in the CO_2_-free incubator at 37 °C. Meanwhile, working solutions of glucose, oligomycin and 2-deoxyglucose (2-DG) were prepared. After another 20 min (50 min incubation of the cartridge) the reagents were added in the ports in working conditions of 1 mM glucose, 1 µM of oligomycin and 50 mM of 2-DG. After one hour of incubation in the new medium, cells were analyzed in the Agilent Seahorse HS Mini Analyzer (*Agilent Technologies*). After each Seahorse experiment a normalization to the total protein amount was performed. The medium was removed from the Seahorse cell plate and 20 µL Lysis-Buffer (10 mM Tris with 0.1% Triton X-100) were added to each well. Lysis of the cells was performed for at least 15 min at room temperature shaking. During lysis Bradford Reagent (*Bio-Rad*, #500–0006) was prepared in water (3:10). A standard dilution was created using a BSA stock (2 µg/µL) and lysis-buffer of concentrations of 0, 0.05, 0.10, 0.15, 0.20 and 0.25 µg/µL. After mixing and centrifuging the standard samples well, 20 µL of each standard was pipetted into a 96-well flat bottom plate. Culture plates were taken from the shaker and 180 µL of the Bradford Reagent was added into each well. Liquids were mixed well by pipetting to remove all attached cells from the plate, and the 200 µL mixture was pipetted into the 96-well at the bottom plate individually. Then 180 µL of the reagent was added to the standards and mixed well by pipetting. Emerged air bubbles were removed before measurement. Optical density was measured at 595 nm in 10 kinetic cycles for 1 min each with a photometer. Protein concentration was estimated via the standard curve and served to normalize the Seahorse data.

### Assessment of mitochondrial content via RT-qPCR

Extraction of the genomic DNA was done according to the vendor's SOP of the *PureLink™* Genomic DNA Mini Kit (*Invitrogen*, K1820-00). For extraction, cells were collected, counted and aliquoted in cell number 5 × 10^6^ cells per tube. The cells were centrifuged at 300 × g for 5 min and resuspended in 200 µL PBS. 20 µL of Proteinase K and RNase A (both 20 mg/mL) were added to the cell suspension, briefly mixed, and incubated for 2 min at room temperature. Afterward, 200 µL of *PureLink™* Genomic Lysis/Binding Buffer were added and mixed well until a homogeneous solution was gained. To promote protein digestion, the solution was then incubated at 55 °C for 10 min. After 10 min, 200 µL of 100% ethanol was applied and a homogeneous mixture was obtained by shaking. The lysate was then moved in a *PureLink™* Spin column in a collection tube and centrifuged at 10,000 × g for one minute at room temperature. The collection tube was then discarded, and the column was placed in a clean *PureLink™* collection kit tube. 500 µL of *PureLink™* Wash Buffer 1 were added to the column and centrifuged again at 10,000 × g for one minute at room temperature. The supernatant and the collection tube were then discarded once more, the column was placed in a new collection tube, and 500 µL of *PureLink™* Wash Buffer 2 were added. Another centrifugation for three minutes at maximum speed at room temperature was executed, the collection tubes were disposed of afterward, and the column was applied in a 1.5 mL sterile microcentrifuge tube. To elute the purified DNA 50 µL of *PureLink™* Genomic Elution Buffer were pipetted in the column, incubated for one minute at room temperature and centrifuged at maximum speed for one minute. To recover more DNA the last step was repeated with the same amount of elution buffer. Collected gDNA was then measured via* NanoDrop* and aliquoted to 20 ng/µL. Expression of two mitochondrial genes (Cox1 and ND4) compared to the expression of a nucleoli gene (B2M) was performed via qPCR (B2M: Fwd: 5´-GTGCTCGCGCTACTCTCTCT-3´, Rev: 5´-TCTCTGCTGGATGACGTGAG-3´; Cox1: Fwd: 5´-TACGTTGTAGCCCACTTCCACT-3´, Rev: 5´-AGTAACGTCGGGGCATTCCG-3´; ND4: Fwd: 5´-ACTCtricarboxylic acidCTGCCCAAGAACT-3´, Rev: 5´-GTGTGAGGCGTATTATACCA-3´). For each qPCR, master mixes were prepared the same way, including 3.6 µL RNase free water (*SIGMA*, W4502), 10 µL SYBR green (*Vazyme*, Q111-02-AA), 0.4 µL ROX-Dye (*Vazyme*, Q111-02-AB) and 0.5 µL of the forward and reverse primer each per well. In total 15 µL of master mix and 5 µL of sample were added to each well of a 96-well plate (*Biozym*, 712,390), respectively. After closing the plate with an optical foil (*Biozym*, 712,350), the plate was centrifuged and put into the light cycler *QuantStudioTM 7 Flex System* (*Thermo-Fisher*, #278,871,692). The denaturation was performed at 95 °C for 3 min, annealing at 59 °C for 45 s and the extension at 72 °C for 7 min, in a repeat of 40 cycles. Data was analyzed using the ΔΔCt-method^55^: ΔCt was determined as the Ct value of a nuclear gene minus the Ct value of a mitochondrial gene (ΔCt), for each possible combination individually, and then averaged. The mitochondrial copy number was computed using the Eq. 2^-ΔCt^.

### Assessment of gene regulation via RT-qPCR

Cells were cultured in T175 flasks until they reached 80% confluency. Subsequently, the cells were harvested and counted as outlined previously. RNA extraction was conducted using the RNeasy Kit (*Quiagen*) following the manufacturer's protocol. The concentration and quality of the isolated RNA were assessed using Nanodrop. cDNA synthesis was performed utilizing 1 ng of RNA and the cDNA Synthesis kit (*ThermoFisher*, K1622), following the manufacturer's instructions. Following cDNA synthesis, 100 ng of cDNA was utilized for RT-qPCR. Experiments were conducted using the *QuantStudioTM 7 Flex System (ThermoFisher*, 278,871,692). Data analysis was performed using the ΔΔCt-method^55^. All RT-qPCR data, primer sequences, and documentation are available in the supplementary material provided.

#### Statistics, and graphs

Statistical comparisons of all data were conducted using unpaired two-tailed Welch’s t-test. Graphs presented in all Figures were generated using GraphPad Prism software. Stars in the figures represent p-values as follows: * for *p* ≤ 0.05, ** for *p* ≤ 0.01, and *** for *p* ≤ 0.001. Unless otherwise stated, standard deviations (SD) are represented by error bars, while the standard error of the mean (SEM) is provided for other experiments.

## Results and discussion

### Overexpression of FAHD1 in U2OS

**Figure 1:**
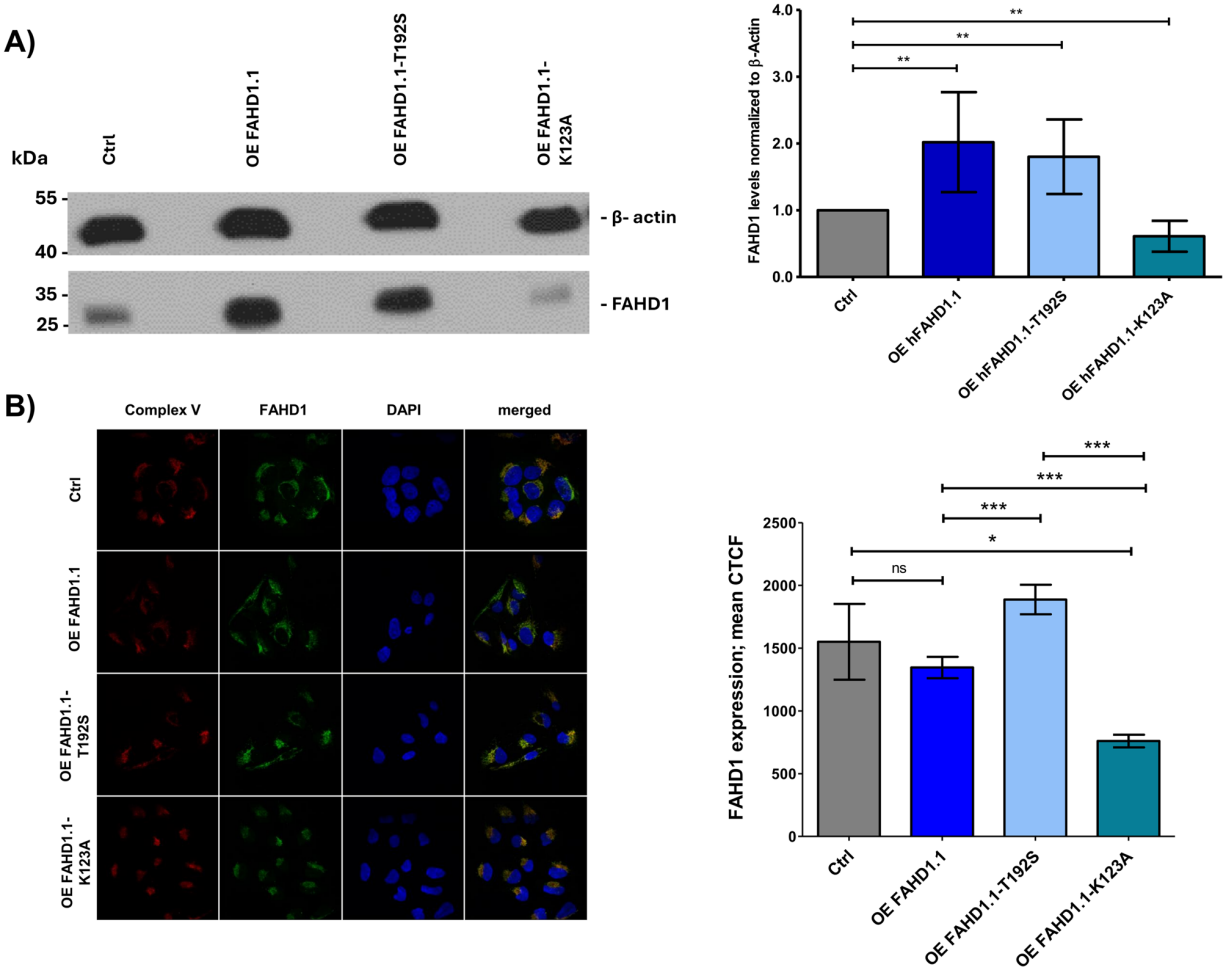
Overexpression of hFAHD1.1 in U2OS. (**A**) Left: An exemplary Western blot analysis was conducted to detect β-actin and hFAHD1 in four U2OS cell lines. It was observed that all FAHD1 bands ran at the same molecular weight, and any apparent shift detected was attributed to gel drift. Right: Quantification of hFAHD1 protein regulation. Protein levels were analyzed using ImageJ, quantifying hFAHD1 levels relative to β-actin. Data were normalized to control cells and represent four biological replicates. Error bars in the graph represent the standard error of the mean (SEM). (**B**) Left: Immunofluorescence staining of three cell lines overexpressing hFAHD1 compared to the control. Right: The corrected total cell fluorescence (CTCF) of hFAHD1 for each cell line was quantified using ImageJ software. The data presented include n = 3 biological replicates, with a minimum of 300 cells analyzed per replicate. Error bars in the graph represent the standard error of the mean (SEM).

It is currently unclear whether FAHD1 may have any non-catalytic functions, such as regulatory roles in signaling pathways. To investigate whether FAHD1 overexpression is attributable to its catalytic activity or simply its expression, mammalian expression vectors containing truncated human promoter regions were utilized (as described in §2.2). A control vector was obtained from *VectorBuilder*, and untreated U2OS cells of comparable passage were used as controls. Following transfection, stable cell lines were obtained through selection (as detailed in §2.2). Protein expression was confirmed via Western blot and RT-qPCR (see Fig. 1 and Fig. S5). U2OS cells typically exhibit low endogenous FAHD1 expression, which was slightly enhanced upon overexpression using the human promoter mimic, as expected. Similar results were observed for the T192S variant. However, to our surprise, overexpression of the K123A variant led to a downregulation of observed FAHD1 protein levels. Since untagged protein was used for transfection, the percentage composition of these bands cannot be determined, i.e., whether the K123A variant shows only endogenous protein or a mixture of endogenous WT and exogenously expressed hFAHD1. In contrast, mRNA levels were significantly upregulated (see Fig. S5), confirming the successful overexpression attempt. Further investigations are needed to elucidate these findings.

**Figure 2:**
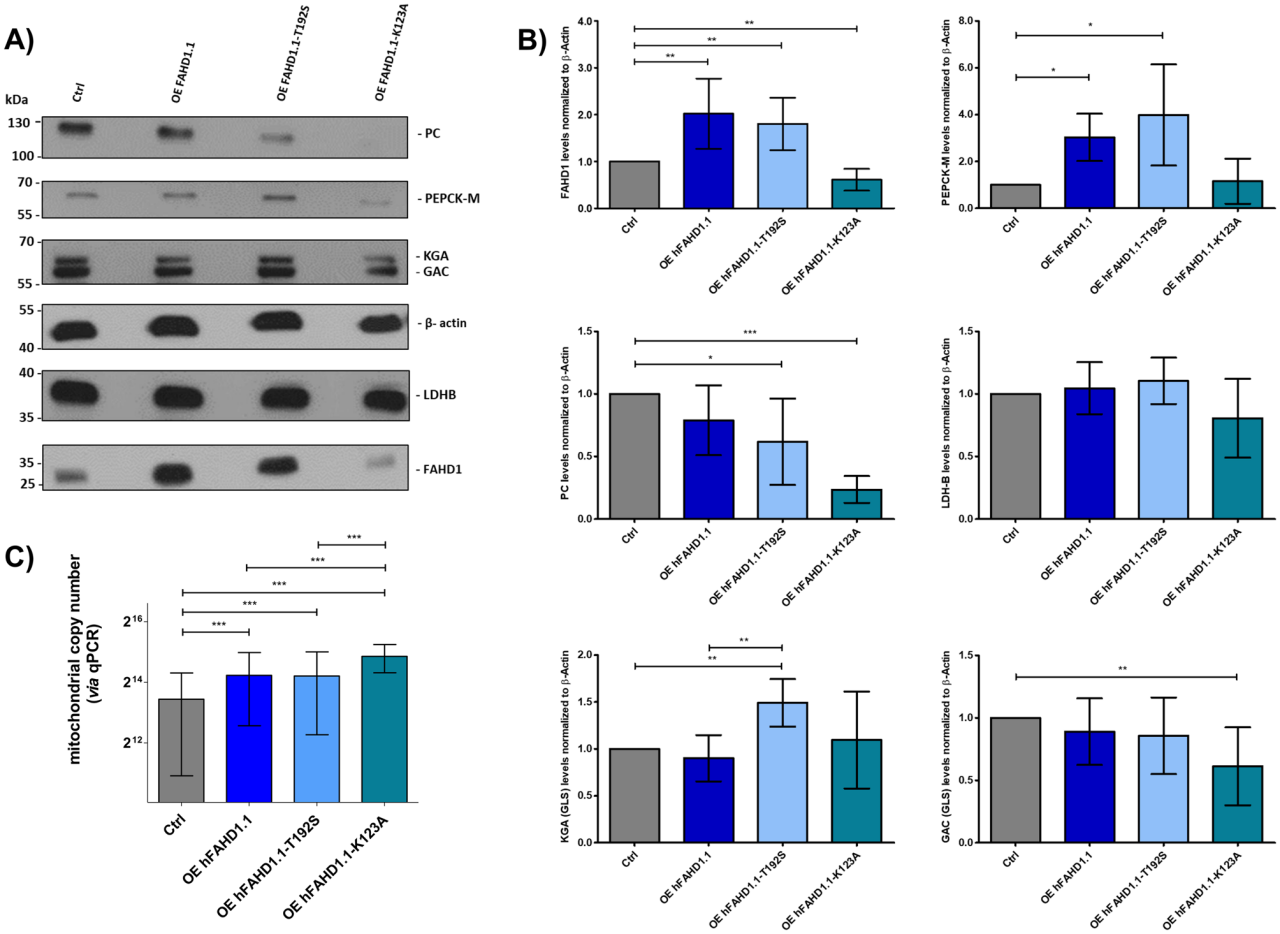
Western blot analysis of protein regulation.** (A**) Exemplary Western blot data is presented. It's important to note that all PC and FAHD1 bands run at the same molecular weight. Any apparent shift observed in the figure is attributed to gel drift. (**B**) Protein regulation was quantified relative to β-actin expression in the same sample and normalized to control cell data. The results are derived from three biological replicates and are presented as mean values ± SEM. (**C**) Mitochondrial copy numbers of cell lines were obtained via qPCR, calculated using ΔCt based on the nuclear marker B2M and the mitochondrial markers ND4 and COX1. Error bars represent the standard error of the mean (SEM) from four biological replicates, each consisting of three technical replicates.

It was observed that PC, mitochondrial PEPCK-M, and GLS (KGA, GAC) are regulated on the protein level (see Fig. 2). Of note, PEPCK-M is upregulated, while PC levels decrease. According to our model, this is a direct consequence of higher mitochondrial pyruvate levels as FAHD1 activity is increased. It was recently reported that the PEP-PYR-OAA node is a key regulator in maintaining mitochondrial metabolism^47^. PEP serves as a precursor for aromatic amino acids, while PYR serves as a precursor for alanine, valine, leucine, isoleucine and lysine, and is important for fermentation pathways that create NAD^+^. However, levels of mitochondrial *lactate dehydrogenase B* (LDH-B) are not regulated. oxaloacetate may react with acetyl-CoA or may be reduced to malate, acting also as a precursor for aspartate, which in turn is used for the biosynthesis of other amino acids, and nucleotides^47^. Connected to this, the *malic enzyme* (ME) couples mitochondria with aerobic glycolysis, especially in osteoblasts^49^. Of interest, previous studies on FAHD1 knockdown in breast cancer cells also observed regulations in GLS^22^. While a downregulation of KGA was observed in U2OS cells overexpressing the FAHD1 wildtype, an upregulation was noted in U2OS cells overexpressing the T192S variant. A similar trend was observed for GAC, although it was not statistically significant. This Western blot data provided a first hint towards a model, where FAHD1 may contribute to the PEP-PYR-OAA node (see §1.6). U2OS cells that overexpress the K123A variant provided a rather unexpected pattern, where PC levels almost vanish, while PEPCK-M and FAHD1 levels are reduced. Upon initial observation, no obvious morphological difference was detected between control cells and T192S/K123A expressing variants in terms of growth/cluster behavior or morphology. This strongly suggests that a metabolic rewiring may be induced to compensate for the altered FAHD1 activity.

### Proliferation in different media conditions

**Figure 3:**
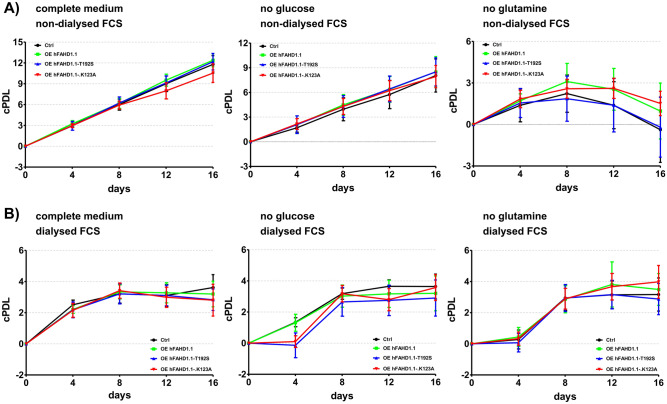
Proliferation of U2OS cell lines in defined media conditions. (**A**) Cumulative population doubling levels (cPDL) of the three U2OS cell lines overexpressing hFAHD1 were compared to control cells under both complete medium conditions and glucose/glutamine deprived conditions. Cells were cultured in 6-well plates with three technical replicates, and counting was performed every 4 days using a Bio-Rad TC20 automated cell counter. Error bars indicate the standard deviation (SD). (**B**) The same series of experiments as depicted in (A) were conducted using dialyzed FCS in all media. The absence of glucose and glutamine from FCS resulted in an altered response in U2OS cells, as they rely on these nutrients for optimal growth conditions. Under these conditions, cell growth was generally comparable to the no glutamine conditions observed with non-dialyzed FCS (**A**).

The selected and verified cell lines were investigated with regard to their proliferation in different defined media conditions (see Fig. 3). In order to prevent cells from reaching confluence prematurely, cell counts were conducted every fourth day.

Osteosarcoma, a prevalent primary bone cancer with a grim prognosis, is identified as a glutamine-dependent cancer type^56,57^. Growth curve analysis reveals that both control cells and the T192S variant (believed to enhance glutaminolysis) exhibit intolerance to glutamine deprivation, whereas cells overexpressing FAHD1 and the K123A variant appear to develop a modest tolerance to glutamine withdrawal. Interestingly, this response differs with glucose; while many cancer cells heavily rely on glucose and cannot survive without it, recent research highlights the impact of glucose and glutamine availability on the metabolism and proliferation of U2OS cells^56^. These findings underscore a more significant role of glutamine, as opposed to glucose, in the growth of U2OS cells, suggesting that glucose exerts limited influence on the proliferation of this particular cancer cell line.

Previous studies have highlighted the significant influence of serum-supplemented metabolites on experimental outcomes. Therefore, we conducted experiments using both non-dialyzed FCS and dialyzed FCS to address this concern. Throughout the experiments, essential growth factors and other critical components from FCS were maintained, ensuring controlled cell proliferation and sustained nutrient availability without reliance on traditional serum components like glutamine or glucose. However, it's important to note that during dialysis, molecules beyond glucose and glutamine, such as uridine, are also removed from the serum. Thus, the observed differences in growth could be attributed to the absence of these vital nutrients. It's crucial to account for such effects to elucidate specific metabolic regulations, a principle we have previously investigated in another study^22^. The remaining glucose the default serum is in part sufficient to sustain proper cell growth in U2OS, which is not the case with dialyzed serum, while withdrawal of glutamine is not tolerated.

**Figure 4:**
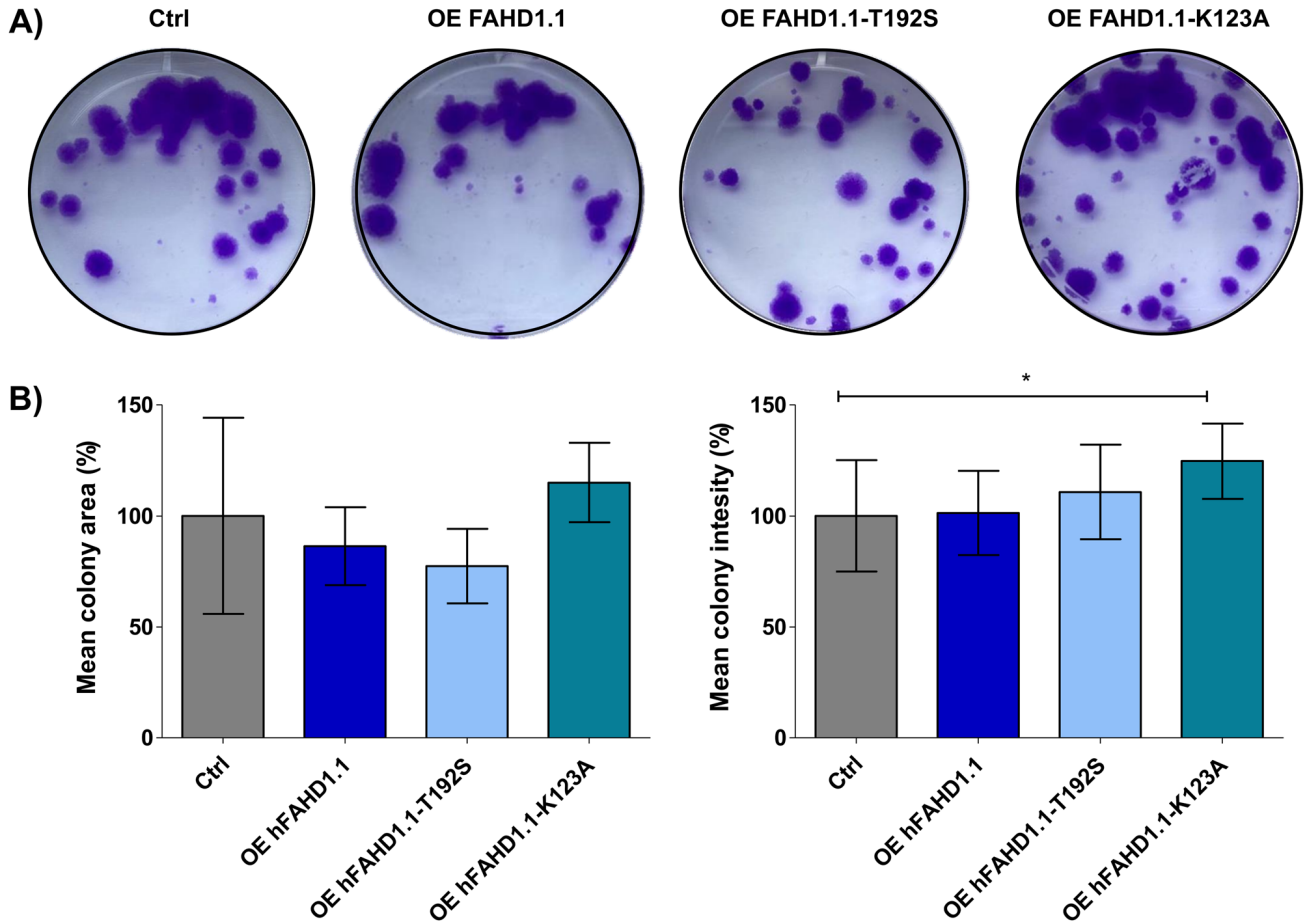
Colony forming assay with U2OS cell lines.** (A**) Cells were seeded in triplicates (100 cells/well in 6-well plates), cultured for 15 days, and subsequently stained with crystal violet. Representative images exhibit one selected well out of three biological replicates that most accurately depict the results. (**B**) The mean area and mean colony intensity covered by colonies are expressed as percentages. Mean colony intensity correlates with the number of cells in colonies. Error bars represent the standard error of the mean (SEM) of three biological replicates, with each replicate performed in three technical replicates. Each well was individually analyzed using the *ImageJ* software with the *ColonyArea* plugin.

The migration ability of U2OS was also tested using a defined scratch assay overnight (data provided as part of the supplementary information). It was found that the migration of U2OS cells overexpressing the FAHD1 T192S variant was comparable to control cells, whereas U2OS cells overexpressing the FAHD1 K123A variant exhibited slightly slower migration. In a similar experiment, a colony formation assay was conducted, as defined^58^. Cells overexpressing hFAHD1.1 and hFAHD1-T192S form comparable amounts of colonies to control cells, with similar migration patterns. Conversely, hFAHD1.1-K123A cells exhibit a significant decrease in colony formation (see Fig. 4).

### Glycolytic flux, ROS levels, and mitochondrial content.

**Figure 5:**
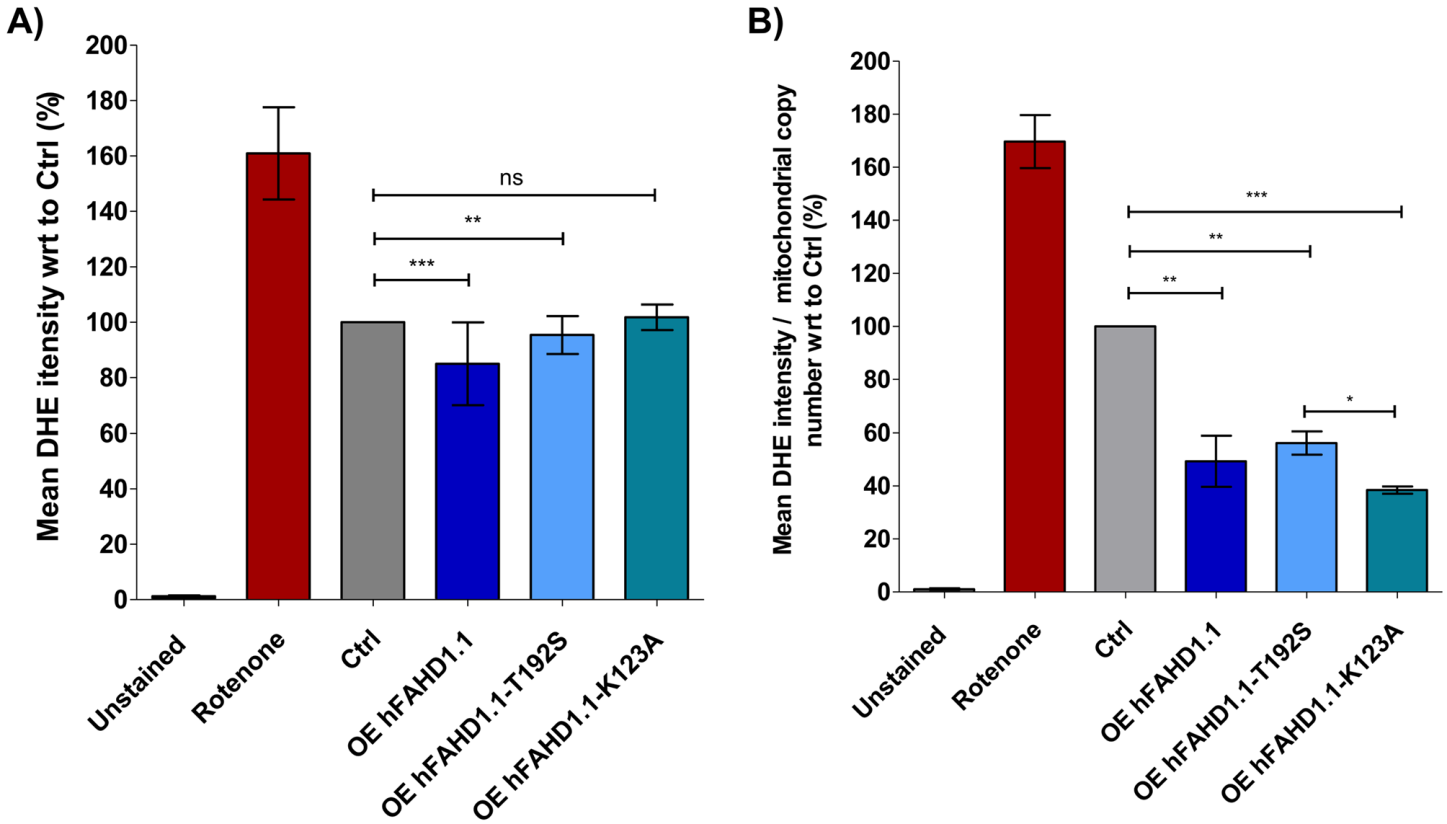
Total cellular ROS accumulation in U2OS cell lines.** (A**) ROS regulation was evaluated by measuring the intensity of 20 µM dihydroethidium (DHE) using flow cytometry. Each data point represents four biological replicates with three technical replicates each, with 3 × 10^5^ cells per tube. In each experiment, a positive control (500 nM rotenone) and an unstained negative control were included. The data were normalized to control cells, and error bars represent the standard error of the mean (SEM). Mean DHE intensity was analyzed using *FlowJo* software. (**B**) The data from panel (A) were normalized to mitochondrial content, as determined via quantitative PCR (qPCR).

The hypothesis posited that overexpressing hFAHD1.1 and hFAHD1.1-T192S in mammalian cells might induce a metabolic shift away from glycolysis, leading to decreased ROS accumulation and cellular proliferation. To explore this hypothesis, ROS levels and mitochondrial content were evaluated (see §2.5 and §2.8). Both hFAHD1.1 and hFAHD1.1-T192S overexpression resulted in decreased intracellular ROS levels and increased mitochondrial content compared to control cells, with the wild-type protein showing a more pronounced effect (see Fig. 5). In contrast, no differences were observed with the K123A variant. However, these differences were attenuated when normalizing the data to mitochondrial content, as determined by RT-qPCR (see §2.8). This indicates that irrespective of the overexpressed variant, there is a general reduction in mitochondrial ROS levels when FAHD1 is overexpressed in U2OS cells.

**Figure 6:**
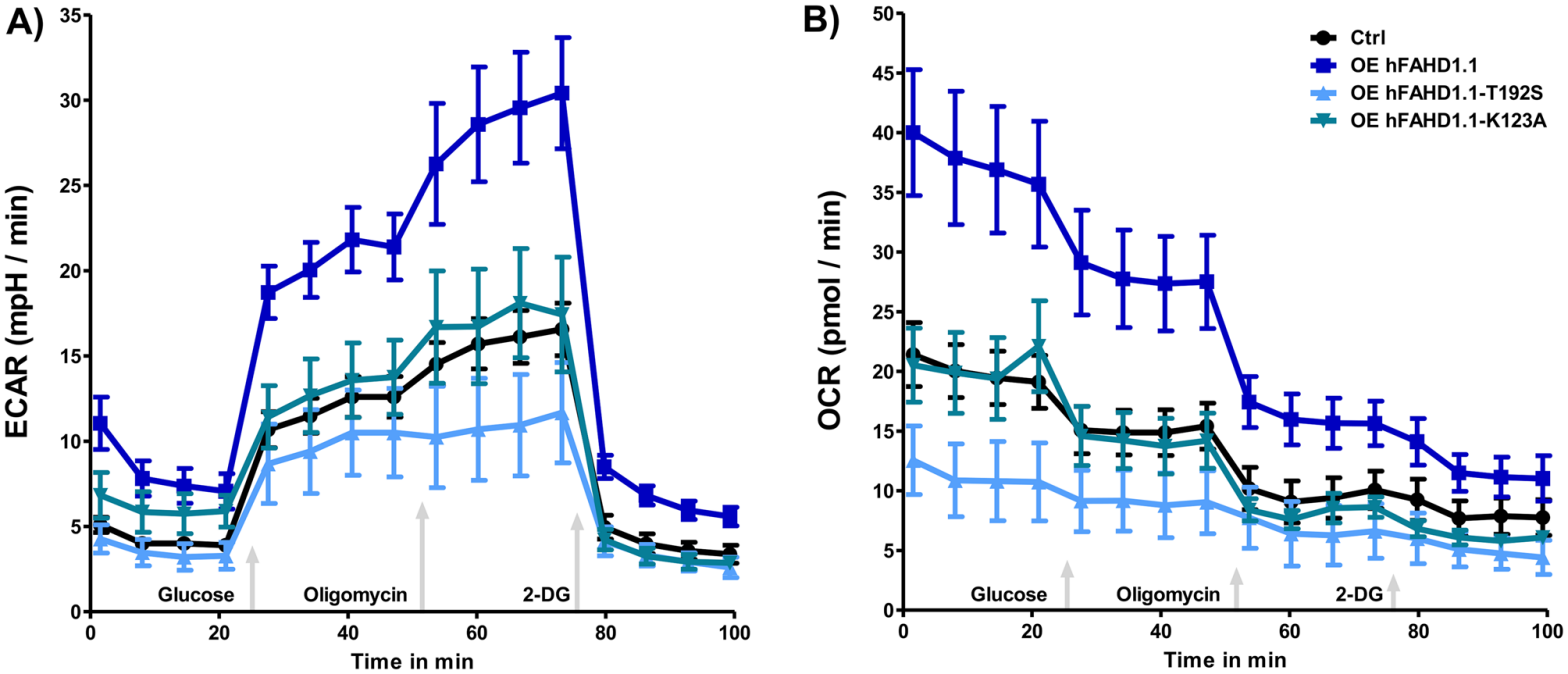
Glycolytic rates in U2OS cell lines. Glycolytic stress test experiments were conducted using a Seahorse XF HS mini with 10^4^ cells/well, seeded one day prior to the experiment. Glucose administration allowed for the assessment of glycolytic function, further potentiated by the inhibition of Complex V using oligomycin (OMY). Subsequently, glycolysis was completely halted by the addition of 2-deoxyglucose (2-DG). Data were normalized to control cells through the evaluation of total protein, determined via Bradford assay after each experiment. Error bars represent the combined standard error of the mean (SEM) from both analyses. (**A**) Extracellular acidification rate (ECAR) (**B**) Oxygen consumption rate (OCR).

**Figure 7:**
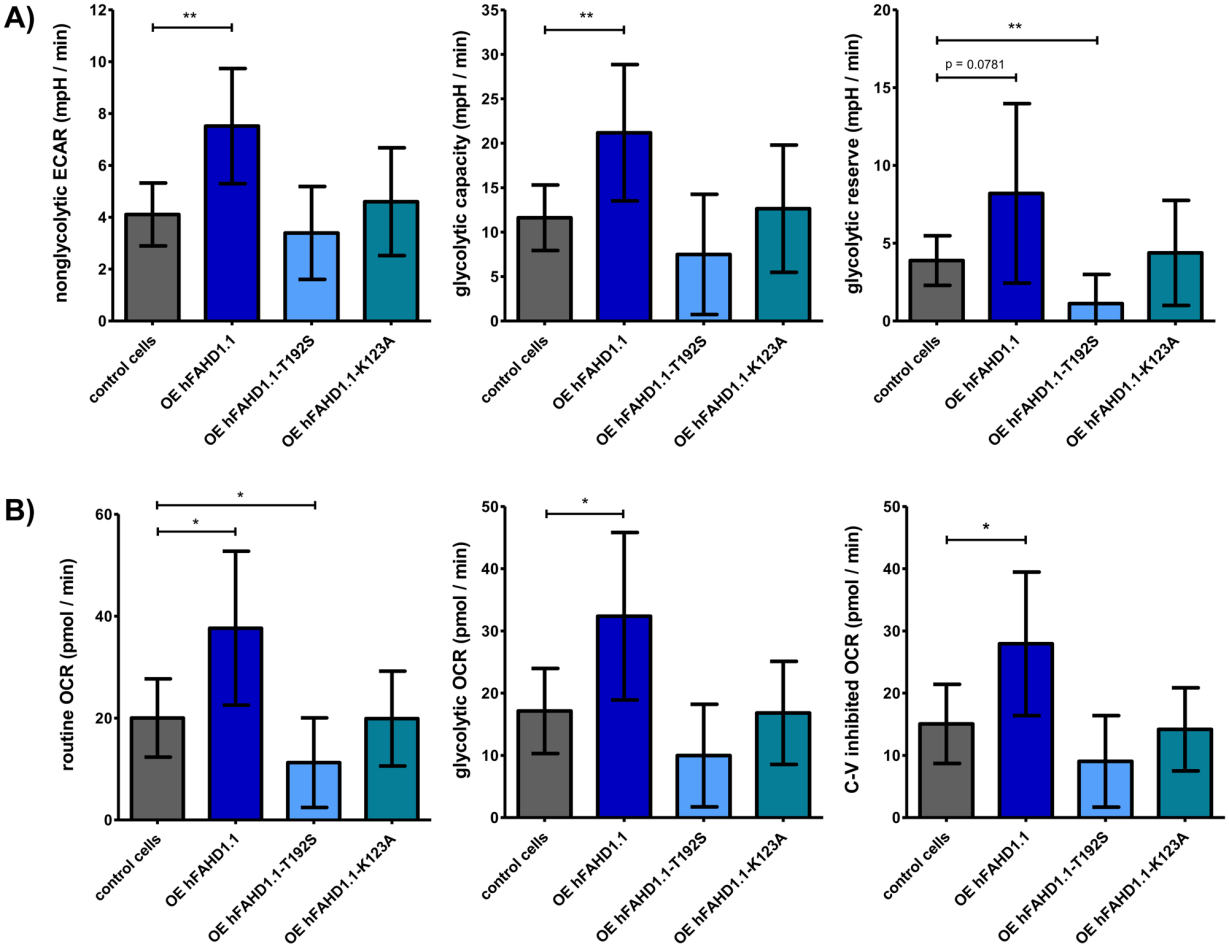
Measurement of glycolytic parameters in U2OS cell lines. Quantification of (**A**) ECAR and (**B**) OCR data obtained by Seahorse *Glycolytic stress test* was conducted following the Agilent glycolytic stress test Standard Operating Procedure (SOP), with error bars indicating standard deviation (SD) (n = 3).

To investigate the glycolytic and metabolic changes upon FAHD1 overexpression in U2OS, glycolysis rate was investigated using Seahorse HS mini flux analysis, accompanied by quantitative Western Blot analysis of relevant protein expression (see Figs. 6 and 7). In hFAHD1.1 overexpressing U2OS cells, an elevated lactate-associated *extracellular acidification rate* (ECAR) was observed compared to control cells. Initially, both hFAHD1.1-T192S and hFAHD1.1-K123A variant expressing cells showed no discernible difference from control cells. However, upon closer examination, it was revealed that hFAHD1.1-T192S expressing cells lack a glycolytic reserve, indicating that these cells are already operating at maximum glycolysis but do not produce lactate, unlike hFAHD1.1 expressing cells. The performance of hFAHD1.1-K123A cells appeared similar to that of control cells; however, Western Blot and RT-qPCR data demonstrated significant downregulation of FAHD1, PC, and PEPCK-M levels in these cells. The associated OCR data indicates a significant decrease in respiration in hFAHD1.1-T192S cells. In contrast, hFAHD1.1-K123A cells exhibit respiration levels comparable to control cells, while hFAHD1.1 expressing cells demonstrate a significantly increased respiration rate.

### Further data analysis and supplementary information

During immunofluorescence experiments on FAHD1 overexpressing U2OS cells, it was observed that both the T192S and K123A variants exhibited a tendency toward misshaped and larger nuclei. However, recognizing the potential for subjective perception to bias such observations, an automated evaluation of nucleus size and shape was sought to be conducted. A pertinent question arising from this approach is whether the cell identity and heterogeneity correlate with its morphology, particularly if the cell-specific proteome correlates with the cell's nucleus morphology. A novel study on *Deep Visual Proteomics* displayed that distinct cell states with individual proteomic profiles may be classified based on imaging, using machine learning techniques^59^. In addressing this question, affirmative answers are provided by Mund et al.^59^, specifically for certain cell types. Deep Visual Proteomics is utilized by them, correlating it to cell nucleus deformation across various cell types, including U2OS cells. Part of this study uses the BIAS software (https://single-cell-technologies.com/bias-2/) to classify the nucleus shapes of U2OS cells based on staining methods. This software, along with the method referenced in literature^59^, was employed to classify the FAHD1 overexpressing U2OS cells using both Hoechst (refer to Fig. S6) and DAPI (refer to Fig. S7) staining. By employing their method of nucleus staining and utilizing their BIAS parameters and evaluation suite (as detailed in their supplementary materials), certain "classes" defined by Mund et al. for U2OS cells based on the nucleus deformation identified in our study were reproduced. The credibility of Mund et al.'s work is implicitly considered, and it is anticipated that the nucleus morphology observed in our cells should, at least in a first-order approximation, correlate with the proteome expression as described by Mund et al. In their supplementary materials, a complete set of genes and their expression levels for each observed "class" of cell types is provided. It is argued that if this correlation holds true, our cells should possess a similar, if not identical, proteome as described by Mund et al. Importantly, it is posited that such regulatory patterns would align well with the model proposed in our manuscript.

Additionally, we obtained preliminary evidence suggesting a potential role of mitochondrial GDP as a cofactor in the FAHD1 catalytic reaction, also aligning with our hypothesis (see Fig. S11). Detailed data regarding these findings can be found in the supplementary materials. While these findings have not yet been corroborated with rigorous experiments, this endeavor is considered a significant aspect of ongoing research and will be included in a future manuscript.

To complement our experiments using Seahorse flux analysis, we also conducted experiments employing high-resolution respirometry (see Fig. S12). Although we were unable to fully uncouple U2OS cells, we did observe indications suggesting an increased glutaminolysis rate in the T192S variant, aligning with our hypothesis.

## Summary

**Figure 8:**
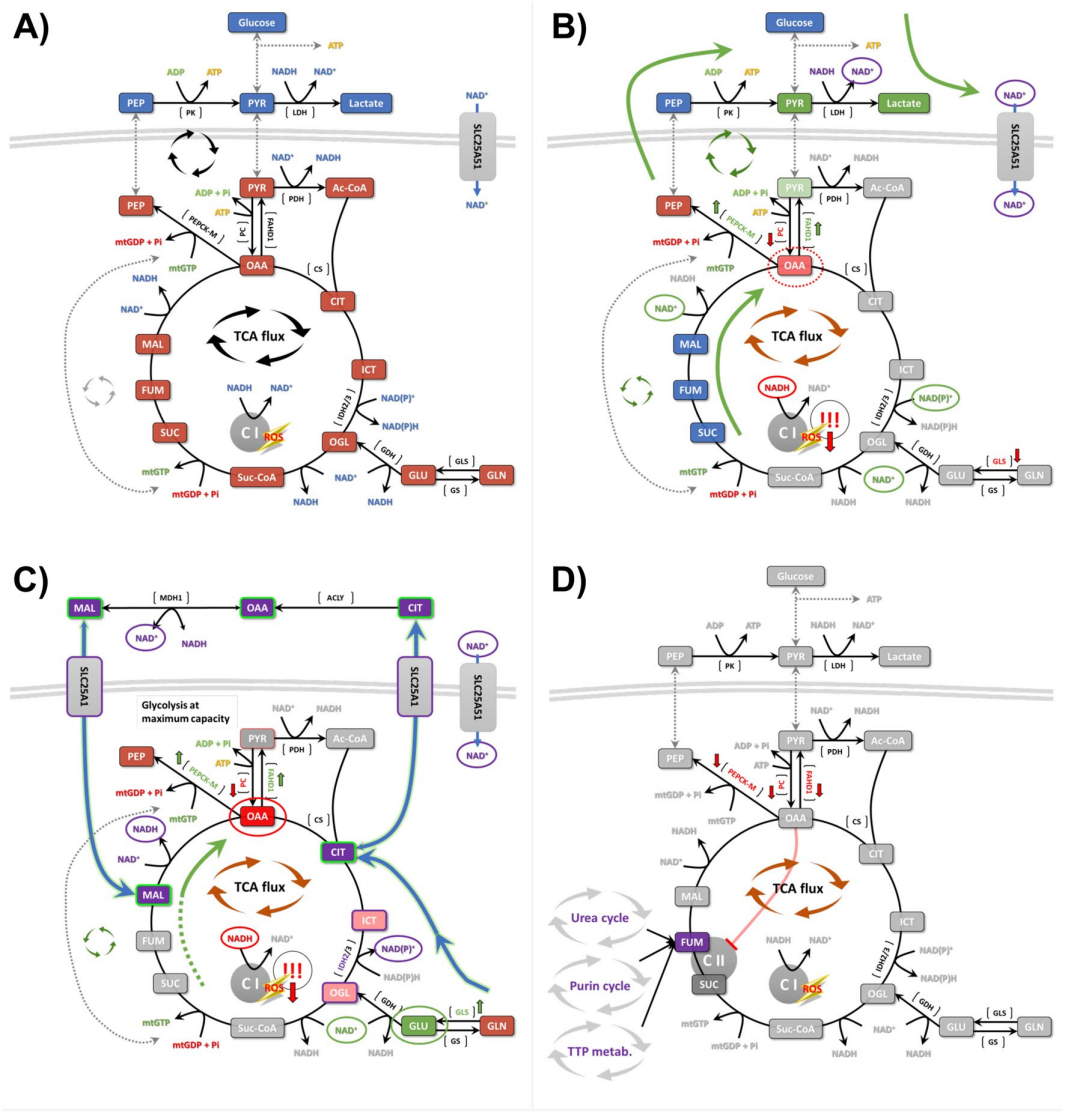
A model of how FAHD1 activity may modulate U2OS metabolism. (**A**) In U2OS cells, the tricarboxylic acid cycle operates independently, relying on both glycolysis and glutaminolysis to sustain the tricarboxylic acid flux. The PEP-PYR-OAA node remains stable, ensuring a consistent supply of mtGDP for the synthesis of succinate (SUC). (**B**) Overexpression of hFAHD1.1 in U2OS cells enhances glycolysis, leading to a reduction in oxaloacetate levels. This reduction impairs the citrate synthase (CS) reaction, consequently diminishing tricarboxylic acid flux and lowering complex I activity and ROS levels. To compensate for oxaloacetate depletion, the PEP-PYR-OAA node is upregulated, generating mtGDP and SUC. With decreased tricarboxylic acid cycle flux, NAD^+^ cannot be produced via complex I, prompting cytosolic lactate production to regenerate NAD^+^. This is facilitated by increased glycolysis. (**C**) Surprisingly, overexpression of hFAHD1.1-T192S in U2OS cells does not enhance glycolysis but shifts metabolism towards glutaminolysis. Similarly to hFAHD1.1 overexpression, oxaloacetate levels are reduced, impairing CS reaction and lowering tricarboxylic acid flux and complex I activity. As oxaloacetate levels may be drastically reduced, the PEP-PYR-OAA node may not sufficiently compensate. Consequently, the tricarboxylic acid cycle may be maintained through glutaminolysis. NADP^+^ may be generated via reductive isocitrate dehydrogenase 2 (IDH2) activity, or NAD^+^ via the nicotinamide (NAM) salvage pathway. Oxaloacetate may also be replenished through a non-canonical tricarboxylic acid cycle, involving citrate export into the cytosol (via SLC25A1), reduction, and subsequent import of malate and NAD^+^ into mitochondria. Malate can then be converted to oxaloacetate, providing fuel for complex I. (**D**) Overexpression of hFAHD1.1-K123A in U2OS cells appears to bypass glycolysis, downregulating PC, FAHD1, and PEPCK-M. This likely indicates a catalytic knockdown of FAHD1 via overexpression of a loss-of-function variant. Although the underlying mechanisms require further elucidation, it seems that glycolysis and the PEP-PYR-OAA node may be completely shut down, with cells relying solely on glutaminolysis. Despite similarities to the wild-type setup (**A**), this metabolic rerouting is weaker. Consequently, these cells exhibit increased mitochondrial content to compensate but otherwise behave similarly to wild-type cells in most experiments, including nearly unchanged ROS levels. Regulation of fumarate (FUM) levels may occur through adjacent pathways to compensate for the lack of biosynthesis.

This work focused on the overexpression of isoform 1 (i.e., hFAHD1.1) in U2OS, suggesting that this introduces a metabolic shift away from glycolysis, thereby reducing ROS accumulation and cellular proliferation. In addition to hFAHD1.1 overexpression via the hFAHD1 promoter, stable cell lines were generated overexpressing the hFAHD1.1-T192S and hFAHD1.1-K123A variants. The T192S variant has increased ODx activity, while the K123A variant is a loss-of-function, but otherwise structurally intact enzyme^13^. These data further suggest a regulatory role of FAHD1 in the PEP-PYR-OAA node^47^. A graphical summary of our hypothesis of how FAHD1 may impact metabolic rewiring in U2OS is provided in Fig. 8.

U2OS cells rely both on glucose and glutamine to preserve the tricarboxylic acid cycle flux, however, with a preference for glutaminolysis^56,57^ (see Fig. 7A). In this state, the PEP-PYR-OAA node works in routine and provides mtGDP to sustain the synthesis of succinate (SUC). NAD^+^ levels are balanced and complex I is active, for which it also creates a certain amount of ROS. According to our model, overexpression of hFAHD1.1 should decrease levels of mitochondrial oxaloacetate (see Fig. 7B). This would lead to a reduction of the tricarboxylic acid flux, as CS is limited in its reaction. As a result, NAD^+^ levels decrease, and complex I cannot work properly anymore, also resulting in decreased ROS levels. It is believed that the metabolism responds to this by upcycling the PEP-PYR-OAA node, leading to an increase in mtGDP-mediated synthesis of SUC. This would explain why these cells show such a high ECAR when exposed to fresh glucose, as increased glycolysis coupled to lactate production will make up for the reduced NAD^+^ levels. Of interest, if this bias is exaggerated by introducing the catalytically enhanced hFAHD1.1 variant T192S, it is believed that the PEP-PYR-OAA node is not anymore able to make up for the lack in NAD^+^, for which a metabolic rewiring happens that shifts the tricarboxylic acid cycle towards glutaminolysis (see Fig. 7C). This may also explain why the T192S cells do not respond to glucose in Seahorse ECAR experiments, and also why they do not display any glycolytic reserve. It is hypothesized that these cells are already operating the glycolysis branch of their tricarboxylic acid cycle at maximum capacity but are unable to generate NAD^+^ through these pathways. Instead, an upregulation of GLS is observed, which is associated with an increased formation of 2-oxoglutarate (OGL). Albeit a reductive tricarboxylic acid cycle is not well demonstrated for eukaryotic cells, our model suggests that the IDH2 reaction may run in reverse, to create NADP^+^ (instead of NAD^+^, as the IDH3 reaction cannot run in reverse)^60^. Alternatively, NAD^+^ may be generated via the mitochondrial NAM salvage pathway (NAMPT/NMN/NMAT2)^61^. If also the CS reaction may run in reverse cannot be stated, however, this may also replenish oxaloacetate levels. This is a phenomenon otherwise known in bacteria and plants. oxaloacetate may also be replenished via a non-canonical tricarboxylic acid cycle^62^, where levels of malate are replenished via export of citrate into the cytosol (SLC25A1), followed by its reduction, and import of malate and NAD^+^ into the mitochondria. Malate may then be converted to oxaloacetate at the gain of NADH, which may fuel Complex I. The PEP-PYR-OAA node still creates mtGDP to create new SDH, but less efficiently than in control cells and hFAHD1.1 expressing cells. The overexpression of the hFAHD1.1-K123A variant leads to several unexpected phenomena, notably the downregulation of PC, PEPCK-M, and FAHD1 itself, despite observing increased mRNA levels consistent with overexpression (see Fig. 7D). Of note, these cells behave similarly to control cells in many respects and are unable to differentiate based on growth and morphology. The data suggests that overall ROS production in these cells remains unchanged compared to control cells. This may be due to the potential bypass or downregulation of glycolysis pathways, prompting these cells to rely solely on glutamine, possibly for fumarate (FUM) formation via alternative routes. The reason for the lack of GLS upregulation in these cells is debatable but holds significant implications for metabolic rewiring.

Moreover, cells overexpressing FAHD1 demonstrate alterations in mitochondrial copy number in reaction to changing conditions. Assessing mitochondrial content is pivotal in metabolic research, providing understanding into cellular energy metabolism and mitochondrial function. Remarkably, discrepancies in ROS levels and other data are only apparent when normalized to mitochondrial copy numbers. Similar findings were observed when examining the impact of FAHD1 depletion in breast cancer cells^22^. Consequently, our data indicates that cells compensate for mitochondrial disruptions resulting from either the loss or elevation of FAHD1 levels by increasing mitochondrial numbers.

Regarding the regulation of GLS, in humans, there are two crucial isoforms of glutaminase (GLS): kidney-type glutaminase (KGA) and glutaminase C (GAC). While KGA is primarily found in the kidney, GAC is distributed throughout various tissues like the brain, liver, and pancreas. These isoforms play essential roles in cellular metabolism by converting glutamine into glutamate, a key precursor for energy production and biosynthesis pathways. GAC, particularly, is implicated in cancer metabolism and is often overexpressed in cancer cells to fulfill heightened energy and biosynthetic requirements. Understanding the functions and regulation of these isoforms is critical for deciphering their roles in normal physiology and diseases like cancer. The distinct localization and regulation of KGA and GAC isoforms significantly impact cancer metabolism, influencing processes such as glycolysis and glutaminolysis. In cancers like osteosarcoma, both isoforms are involved, with heightened expression correlating with increased glycolysis and glutaminolysis, driving tumor growth and metastasis.In the hFAHD1.1 wild-type overexpressing cells, a downregulation of KGA protein (as observed through Western Blot analysis) was noted, whereas in the T192S samples, an upregulation was observed. Similarly, a comparable trend was observed in the GAC samples, although it did not reach statistical significance. These observations align with prior research findings that elevated KGA expression has been associated with enhanced cell growth and migration^63^, and that hypoxic conditions have been shown to increase GLS levels, indicating a potential role in adaptive responses to low oxygen environments^64^.

In summary, our initial hypothesis regarding the overexpression of FAHD1 and its T192S variant in mammalian cells, potentially inducing a metabolic shift away from glycolysis, thereby reducing ROS accumulation and cellular proliferation, was strongly supported by our experimental findings. Although conducted in U2OS cells, our data may hold broader relevance to the global scientific community, particularly considering that the T192S variant of FAHD1 is commonly expressed across various animal species. As discussed in the introduction, this variant is particularly prevalent in animals exhibiting heightened resistance to oxidative stress and a propensity for longevity. Conversely, the findings regarding the K123A variant prompt intriguing questions regarding the potential role of FAHD1 in mitochondrial metabolism, including metabolic alterations, calcium homeostasis, and cell development. Targeting glutaminase activity emerges as a potential therapeutic strategy for osteosarcoma and other cancers, aiming to disrupt metabolic dependencies and hinder tumor progression. Understanding the possible regulation of KGA and GAC isoforms by FAHD1 regulation may help to identify potential therapeutic targets for cancer treatment. Further investigation of these aspects is actively pursued, and additional findings will be presented in due course.

### Supplementary Information

Supplementary Figures.

## Data Availability

Exemplary data files and full statistical data evaluation are part of the supplementary material. Full datasets generated and/or analyzed during the current study are available from the corresponding author upon reasonable request.
