# Supplementary material for: Mitochondrial enzyme FAHD1 reduces ROS in osteosarcoma
Source: Sci Rep. 2024 Apr 22;14:9231. doi: 10.1038/s41598-024-60012-x (PMC11035622; doi:10.1038/s41598-024-60012-x)
Supplement: Supplementary file 1 — Supplementary Figures. [file 41598_2024_60012_MOESM1_ESM.docx]

**Supplementary Information**

**Mitochondrial Enzyme FAHD1 Reduces ROS in Osteosarcoma**

Heberle^1^, Anne; Cappuccio^1^, Elia; Andric^1^, Andreas; Kuen^1^, Tatjana; Simonini^1^, Anna; Weiss^1,^**^*^**, Alexander K. H.

1) University of Innsbruck, Institute for Biomedical Aging Research

*) corresponding author

In this supplementary file, we present a comprehensive summary of our experimental data and statistical analyses. Organized **alphabetically by methods**, as outlined in the subsequent **table of contents**, this supplementary material serves as a repository for all pertinent data generated throughout our study. By centralizing this information, we aim to facilitate access to detailed experimental procedures and results, thereby enhancing the transparency and reproducibility of our research findings.

All **Supplementary Figures are labeled according to their reference points in the main text**; therefore, the numbering in this file may differ from the alphabetical sorting of the methods. It is important to **utilize this supplementary material in conjunction with the main manuscript** to fully comprehend and contextualize the presented data. A **list of Supplementary Figures** is provided to help find the figures while reading the main text.

**Original data files and statistical evaluations** are provided on reasonable request (please contact the corresponding author), as the editorial team of this Journal wanted us to provide a major supplementary file (i.e., this), including only the major Figures and Diagrams.

**Overview:**

[S1) BIAS analysis	4](#_Toc163116732)

[S2) Colony forming assay	10](#_Toc163116733)

[S3) DHE assay FACS	11](#_Toc163116734)

[S4) Docking and *in silico*	12](#_Toc163116735)

[S5) Growth curve	18](#_Toc163116736)

[S6) Immuno-Fluorescence (IF) quantification	19](#_Toc163116737)

[S7) Kinetic assays	20](#_Toc163116738)

[S8) Mitochondrial content	27](#_Toc163116739)

[S9) RT-qPCR quantification	28](#_Toc163116740)

[S10) Scratch assay	31](#_Toc163116741)

[S11) Seahorse and Oxygraph data	36](#_Toc163116742)

[S12) Vectormaps	41](#_Toc163116743)

[S13) Western Blot (WB) quantification	45](#_Toc163116744)

[References:	78](#_Toc163116745)

**List of Figures:**

**Supplementary Figure S1**: comparing FAHD1 to Cg1458 …………………………...	20

**Supplementary Figure S2**: FAHD1-T192S kinetics …………………………..............	21

**Supplementary Figure S3**: *In silico* experiments with FAHD1-T192S ….…………...	13-14

**Supplementary Figure S4**: FAHD1-T192S expression in animals …………………...	16-17

**Supplementary Figure S5**: RT-qPCR gene expression upon FAHD1 OE …………...	29-30

**Supplementary Figure S6**: wound healing / scratch assays …………………………...	32-33

**Supplementary Figure S7**: cell migration assays …………………………………….....	34-35

**Supplementary Figure S8**: BIAS analysis of nucleus morphology (HOECHST) .…...	6-7

**Supplementary Figure S9**: BIAS analysis of nucleus morphology (DAPI) …………...	8-9

**Supplementary Figure S10**: citrate synthase activity data …………………..………...	23

**Supplementary Figure S11**: mGDP may be a cofactor of FAHD1 ……………………	25-26

**Supplementary Figure S12**: high resolution respirometry ……………………………..	37-40

# S1) BIAS analysis

The BIAS software ([*https://single-cell-technologies.com/bias-2/*](https://single-cell-technologies.com/bias-2/)) was utilized for performing machine-learning-driven principal component analysis of nucleus shape stained *via* Hoechst and DAPI staining at both 40x and 60x magnification. A pertinent question arising from this approach is whether the cell identity and heterogeneity correlate with its morphology, particularly if the cell-specific proteome correlates with the cell's nucleus morphology. In addressing this question, affirmative answers are provided by Mund et al.^1^, specifically for certain cell types. Deep Visual Proteomics is utilized by them, correlating it to cell nucleus deformation across various cell types, including U2OS cells. Their analysis program (BIAS), along with the exact parameterization of their machine learning model, adapted for U2OS cell detection, was obtained by us. Parameters for the BIAS analysis of U2OS cell was obtained from the supplementary materials at [*https://www.ebi.ac.uk/biostudies/studies/S-BSST820?query=S-BSST820*](https://www.ebi.ac.uk/biostudies/studies/S-BSST820?query=S-BSST820). By employing their method of nucleus staining and utilizing their BIAS parameters and evaluation suite (as detailed in their supplementary materials), certain "classes" defined by Mund et al. for U2OS cells based on the nucleus deformation identified in our study were reproduced. The credibility of Mund et al.'s work is implicitly considered, and it is anticipated that the nucleus morphology observed in our cells should, at least in a first-order approximation, correlate with the proteome expression as described by Mund et al. In their supplementary materials, a complete set of genes and their expression levels for each observed "class" of cell types is provided by Mund et al. It is argued that if this correlation holds true, our cells should possess a similar, if not identical, proteome as described by Mund et al. Importantly, it is posited that such regulatory patterns would align well with the model proposed in our manuscript. While these findings have not yet been corroborated with PCR experiments, this endeavor is considered a significant aspect of ongoing research and will be included in a future manuscript.

While observing the FAHD1 overexpressing U2OS cells in immunofluorescence experiments, we noted that both the T192S and K123A variants displayed a trend towards rather misshaped and larger nuclei. However, as such observations tend to be biased by subjective perception, we aimed for an automated evaluation of nucleus size and shape. A novel study on *Deep Visual Proteomics* displayed that distinct cell states with individual proteomic profiles may be classified based on imaging, using machine learning techniques ^2^. Part of this study uses the BIAS software ([*https://single-cell-technologies.com/bias-2/*](https://single-cell-technologies.com/bias-2/)) to classify the nucleus shapes of U2OS cells based on staining methods. We used this software and the referenced method^2^ to classify the FAHD1 overexpressing U2OS cells, based on both Hoechst (see **Fig. S8**) and DAPI (see **Fig. S9**) staining. While BIAS analysis with Hoechst staining at 40x magnification did not display strong changes, the same analysis using DAPI staining at 60x magnification provided data, that both the T192S and K123A variants indeed display a different variety of nucleus area and shape, compared to control cells and the hFAHD1 overexpressing cells. As principal component analysis is individual for the given data set, one cannot simply compare clusters among studies, i.e., we cannot compare our data to the data provided in the referenced proteomic study^2^. However, based on this data we may observe certain similarities. In the supplementary data provided by the referenced work^2^, preferred clusters (note: clusters are ranked in type by preference, i.e., cluster 1 is most common, and so on…) show an upregulation of pathways associated with collagen, elastin, and molecules associated with elastic fibers, while less preferred clusters tend to downregulate pathways associated to Golgi-to-ER processes^2^. Concerning proteins, they show upregulation of *Acetyl-CoA acetyltransferase* (ACAT1), *ATP-citrate synthase* (ACLY), *Cell cycle and apoptosis regulator protein 2* (CCAR2), *Cadherin-2* (CDH2), *GTP-binding nuclear protein Ran* (RAN), and *Spermidine synthase* (SRM), among others. They show downregulation of *Citrate synthase* (CS), *Catenin alpha-1* (CTNNA1), *Fatty acid synthase* (FASN), *Kidney-type Glutaminase* (KGA, GLS), *Aspartate aminotransferase* (GOT2), *Hexokinase-1* (HK1), *Nicotinamide phosphoribosyltranferase* (NAMPT), *D-3-phosphoglycerate dehydrogenase* (PHGDH), *Calcineurin subunit B type 1* (PPP3R1), and *Vimentin* (VIM), among others. They show rather unchanged levels of *L-Lactate dehydrogenase B* (LDHB), *Myosin light chain polypeptide 6* (MYL6), and *Ornithine aminotransferase* (OAT), among others. These patterns in protein regulation correlate with our data, but also imply that introducing an overexpression of hFAHD1.1 and its two variants T192S and K123A in U2OS may also impact other important pathways other than the tricarboxylic acid cycle.

**Supplementary Figure S8: BIAS analysis of U2OS cell lines *via* Hoechst staining. A**) We utilized the BIAS software ([*https://single-cell-technologies.com/bias-2/*](https://single-cell-technologies.com/bias-2/)) to conduct machine-learning-driven principal component analysis of nucleus shape stained with Hoechst. Exemplary images of classified clusters are presented. **B**) The area and eccentricity of nuclei were determined for each cluster and cell line. No significant differences were observed between the cell lines, indicating that the machine-learning algorithm successfully identified similar nuclei in each experimental condition. **C**) The percentage of nuclei classified into specific clusters varied across cell lines. With Hoechst staining at 40x magnification, few differences were observed, but there was an indication of fewer nuclei classified as cluster type 4 in the T192S and K123A cells.

**Supplementary Figure S9: BIAS analysis of U2OS cell lines *via* DAPI staining. A**) Utilizing the BIAS software ([*https://single-cell-technologies.com/bias-2/*](https://single-cell-technologies.com/bias-2/))*,* we conducted machine-learning-driven principal component analysis on nuclei stained with DAPI to classify clusters. Exemplary images of classified clusters are shown. **B**) The area and eccentricity of nuclei were determined for each cluster and cell line. No significant differences were observed between the cell lines, suggesting that the machine-learning algorithm identified similar nuclei in each experimental setup. **C**) The percentage of nuclei classified into specific clusters varied across cell lines. With DAPI staining at 60x magnification, more differences were observed compared to Hoechst staining. Specifically, K123A cells exhibited a higher proportion of nuclei classified as cluster type 2 and a lower proportion of cluster types 1 and 3, while T192S cells showed a higher proportion of cluster type 4 nuclei.

# S2) Colony forming assay

Colony-forming assays were performed using U2OS cells following a previously described protocol ^3^. Cells were cultured in triplicates in 6-well tissue culture plates (*FALCON*, 353046), with 100 cells seeded per well on day 0. The cells were cultured for 15 days with medium changes every four days. On the 15^th^ day, the medium was removed, and cells were washed with PBS (*SIGMA*, D8537-500ML). Formed colonies were fixed in methanol for 5 minutes at room temperature. After fixation, cells were washed with PBS and stained with 0.5% crystal violet solution for 20 minutes at room temperature. Excess staining solution was removed by washing the cells multiple times with ddH_2_O. The wells were left to dry, and the area covered by colonies, as well as the intensity of the colonies, were determined using the *ColonyArea* plugin in the *ImageJ* software^4^. **Original data files and statistical evaluations** are provided on reasonable request.

# S3) DHE assay FACS

A group of experts has proposed guidelines for measuring ROS in cultured cells^5^. The referenced recommendation emphasizes using DHE or MitoSOX probes with validated specific products, minimizing probe concentration, and incorporating controls for membrane potentials. LC–MS methods are suggested for precise measurement of all modified species. It's worth noting that our fluorescence staining, and quantification were conducted via FACS experiments rather than imaging. DHE is a commonly used agent in Flow Cytometry for ROS detection. Despite its challenges, stringent controls ensure reliable quantification. Negative controls gauge background fluorescence, while positive controls validate assay sensitivity to ROS. Gating strategies are employed to exclude debris and non-viable cells, ensuring accurate quantification.

Although DHE presents challenges like fluorescence overlap and non-specific oxidation products, meticulous experimental design with appropriate controls facilitates reliable ROS quantification. We designed such controls in this study, as outlined in the “Materials and Methods” section of the main manuscript. Regarding MitoSOX, we are cautious due to its limitations. The method poses challenges such as potential fluorescence misinterpretation caused by both specific and non-specific oxidation products with overlapping spectra, including ethidium and 2-hydroxyethidium. Additionally, factors like membrane potentials and mitochondrial characteristics can influence probe accumulation and fluorescence quenching, potentially affecting measurement reliability. Moreover, MitoSOX oxidation products may intercalate into DNA, leading to fluorescence amplification and artifact creation.

**Original data files and statistical evaluations** are provided on reasonable request.

# S4) Docking and *in silico*

We utilized *AutoDock Vina*^6^ software to dock oxaloacetate into the binding pocket of both the wild-type and mutant proteins (refer to **Fig. S3A**). Although there were only minor changes observed in the overall structure, the absence of the methyl group in the sidechain of S192 potentially allows the hydroxyl group to reorient and face the substrate. Initially, we anticipate that the hydroxyl group of S192 is directed towards the backbone oxygen of an adjacent substructure, rather than facing towards the cavity center, as seen with T192. In the case of T192, we expect the methyl group to hinder the interaction with the carbonyl group of the Mg^2+^-binding substrate, thereby sterically preventing rotation of the T192 sidechain. However, this scenario may differ with S192, where the absence of the methyl group could lead to different interactions. To further investigate this, we conducted quantum chemical simulations using the *QuMuLuS^++^* software (accessible at https://sourceforge.net/projects/qumulus/). The simulations were performed at the Hartree-Fock level with Stuttgart-ECP basis, incorporating point-charge embedding of all relevant Gasteiger charges computed by *AutoDock Vina*^6^ and a polarizable continuum model to compensate for the absence of the protein cavity (refer to **Fig. S3B**).

**Supplementary Figure S3: Virtual docking of oxaloacetate into the cavity of hFAHD1 and its T192S variant. A)** Utilizing the *AutoDock Vina* software suite, we conducted virtual docking of oxaloacetate into the cavity of hFAHD1 and its T192S point mutation. While observing minor alterations in the overall structure, the absence of the methyl group at the sidechain of S192 could potentially allow the hydroxyl group to reorient and face the substrate. However, it's important to note that this is solely a model, and confirmation of this hypothesis would necessitate a crystal structure. **B)** Building upon the docking data of oxaloacetate into the cavity of hFAHD1 and its T192S point mutation, we conducted quantum chemical calculations to compute the electron density in the active site. Our findings reveal that the S192 sidechain is oriented towards the substrate. Additionally, we observed that the electronic density of the Highest Occupied Molecular Orbital (HOMO) state is primarily centered on the Mg^2+^ ion; however, this density is shifted towards the carbonyl and keto groups of oxaloacetate.

Under these conditions, we observe that the S192 sidechain faces the substrate due to the absence of the methyl group, a characteristic that is not present in the T192 variant. While the electron density of core electrons remains unchanged, the mutation induces a significant shift in the electronic density of valence electrons and the Highest Occupied Molecular Orbitals (HOMO state). In the wild-type, electronic density predominantly centers on the Mg^2+^ ion, whereas in the mutant, it shifts towards the carbonyl and keto groups of oxaloacetate. This shift suggests a potential stabilization of the keto-enol tautomerization process, which serves as the initial step towards decarboxylation. Such a model could elucidate the enhanced catalytic activity of the mutant protein and its reduced substrate binding affinity, as observed in wet-lab experiments using the recombinant protein. However, it's important to emphasize that this model remains theoretical, and validation through a crystal structure is essential.

Of significant interest, we noted a surprising correlation between the expression of the T192S variant of FAHD1 in certain animals exhibiting long lifespans and resistance to cancer. This correlation is attributed to a heightened resistance to the detrimental effects of senescence and robust responses to environmental stressors (refer to **Fig. S4**).

**Supplementary Figure S4: The FAHD1 S-variant is preferentially found in long-living animals.** We conducted a BLASTp search for human FAHD1 isoform 1 across all known species. For each species identified, we consulted the *AnAGE* database ([*https://genomics.senescence.info/download.html#anage*](https://genomics.senescence.info/download.html#anage)) to gather information on maximum lifespan and average adult weight. In aging research, a commonly accepted metric for comparing lifespan among species is the ratio of total lifespan to adult weight, expressed as years per kilogram (y/kg). The data presented illustrates the average lifespan/weight of (known) FAHD1-expressing animals, with standard error of the mean (SEM), categorized into T-variant and S-variant groups corresponding to human FAHD1 isoform 1. The figures depict three categories of significance: **A**) This panel displays all available data, excluding two notable outliers: the Little brown bat (*Myotis lucifugus*) with approximately 3400 y/kg, expressing the T-variant, and the King cobra (*Ophiophagus hannah*) with around 3750 y/kg, expressing the S-variant. A discernible trend is observable among certain families of bats and birds, although not all birds carry the S-variant, and not all bats carry the T-variant. **B**) This panel illustrates all data falling below the cutoff of 1000 y/kg. When data below the cutoff of 200 y/kg are examined, they exhibit a level of significance close to one-star (p = 0.00521) (not shown). A general trend towards higher lifespan/weight is evident among animals carrying the S-variant. This trend is particularly noticeable in certain birds, as highlighted in panel A; conversely, it appears that certain long-lived bats express the T-variant.

# S5) Growth curve

Growth curve experiments are a fundamental technique used in biology and microbiology to study the growth dynamics of cells, microorganisms, or populations over time. By monitoring changes in population size or cell density at regular intervals, growth curves provide valuable insights into various biological processes, including cell proliferation, microbial growth, and population dynamics. These experiments typically involve culturing organisms in a suitable growth medium under controlled conditions while measuring growth parameters such as optical density, colony-forming units, or cell counts. Growth curves can reveal essential information about growth kinetics, including lag phase, exponential growth phase, stationary phase, and death phase, shedding light on factors influencing growth rates, metabolic activity, and environmental responses. Moreover, growth curve analysis is widely utilized in diverse fields, including microbiology, cell biology, biotechnology, and pharmacology, to assess the effects of environmental conditions, nutrients, drugs, or genetic manipulations on growth behavior, thereby aiding in the characterization and optimization of biological systems.

**Original data files and statistical evaluations** are provided on reasonable request.

# S6) Immuno-Fluorescence (IF) quantification

Immuno-Fluorescence (IF) quantification is a powerful technique widely used in cell biology and molecular biology to visualize and quantify the expression levels and localization of specific proteins within cells or tissues. This method utilizes fluorescently labeled antibodies to target and bind to the protein of interest, allowing for its detection and visualization under a fluorescence microscope. By quantifying the intensity and distribution of fluorescence signals, IF quantification enables researchers to assess protein expression levels, subcellular localization, and cellular distribution patterns. Moreover, IF quantification can provide valuable insights into dynamic cellular processes, such as protein-protein interactions, post-translational modifications, and changes in protein expression in response to various stimuli or treatments. In this way, IF quantification serves as a powerful tool for elucidating the molecular mechanisms underlying physiological and pathological processes. This introduction sets the stage for the subsequent discussion of experimental data and statistical evaluations of IF quantification experiments. Experiments were conducted following the procedures outlined in the "Materials and Methods" section of the main manuscript. **Original data files and statistical evaluations** are provided on reasonable request.

# S7) Kinetic assays

***FAHD1-T192S:***

A small subset of enzymes within the FAH superfamily exhibit oxaloacetate decarboxylase activity^7,8^. The significance of a human FAHD1 T192S variant was identified through a BLAST search against the highly active oxaloacetate decarboxylase enzyme Cg1458 from *Corynebacterium glutamicum*^9–11^. Although FAHD1 demonstrates unexpectedly low catalytic activity^12^, Cg1458 exhibits remarkably high catalytic efficiency^10,11^. Analysis revealed that while the catalytic regions of Cg1458 and FAHD1 share conservation, they differ at a single position, where S258 of Cg1458 corresponds to T192 in human FAHD1 (see **Fig. S1**).

**Supplementary Figure S1: Comparing hFAHD1 to Cg1458.** Although the primary catalytic sites of human FAHD1 and Cg1458 exhibit remarkable similarity, a comparison of their protein sequences highlights two notable differences. Amino acids crucial for catalytic activity are demarcated by borders. In addition to the amino acids present in the lid structure of Cg1458, which are absent in hFAHD1, we observe that S258 of Cg1458 corresponds to T192 in hFAHD1.

Our kinetic data for the T192S point mutation of human FAHD1, following a protocol similar to that described elsewhere^12,13^, demonstrate enhanced catalytic activity compared to the wild-type protein (see **Fig. S2**). Kinetic data for Cg1458 were obtained from external studies^10,11^. While we observed Michaelis-Menten kinetics with v_max_ = 0.215 ± 0.006 µmol/min/mg and K_M_ = 41.6 ± 5.9 µM for the wild-type protein, the T192S mutant form exhibited v_max_ = 0.740 ± 0.017 µmol/min/mg and K_M_ = 322.7 ± 29.9 µM. To gain further insight into the molecular implications of the T129S variant, we conducted in silico experiments based on the wild-type model.

**Supplementary Figure S2: Kinetic characterization of hFAHD1.1 and its T192S variant. A)** oxaloacetate decarboxylase kinetic assays (n = 3) using purified recombinant proteins were conducted to characterize the Michaelis-Menten kinetics of hFAHD1 and its T192S point mutation. **B)** In contrast to the remarkably high oxaloacetate decarboxylase activity of Cg1458, the wild-type human FAHD1 exhibits surprisingly low catalytic activity. We observed that the human FAHD1 mutation T192S displays both significantly higher v_max_ and K_M_ values compared to the wildtype.

***Citrate Synthase Assay:***

10^6^ cells were aliquoted into 1.5 mL tubes containing growth medium, centrifuged at 250 x g for 5 minutes, and then resuspended in 100 µL of RIPA buffer (for example, see https://www.novusbio.com/support/general-support/buffers.html). The samples were kept on ice for further processing and either frozen at -80°C or in liquid nitrogen for storage. To assess the levels of citrate synthase, a modified protocol based on the procedure provided by Oroboros Instruments was employed ([*https://wiki.oroboros.at/index.php/Citrate_synthase*](https://wiki.oroboros.at/index.php/Citrate_synthase)). After 16 days in culture with dialyzed FCS, we obtained cell lysates for all cells (all lines in all medium conditions). CS activity was highest in control cells at complete medium conditions (i.e., pyruvate, glucose and glutamine), but significantly decreased with hFAHD1.1, hFAHD1.1-T192S, and hFAHD1.1-K123A cells. This is a discrepancy between the increased mitochondrial mass measured with RT-qPCR and the decreased mitochondrial mass indicated by citrate synthase levels. Upon closer examination, we have identified potential factors that could contribute to this discrepancy, including the inherent variability in experimental assays. While RT-qPCR provides insights into mitochondrial DNA content, citrate synthase activity measures mitochondrial enzyme activity, which may not always correlate directly with mitochondrial mass. Additionally, variations in sample processing, assay conditions, and technical limitations could influence the outcomes of these measurements.

**Supplementary Figure S10: Citrate synthase activity in U2OS cell lines.** Cells were cultured for 16 days under defined medium conditions using dialyzed fetal calf serum (FCS). **A**) Citrate synthase activity was normalized to total protein content. Please note that this assay employs citrate synthase activity with a standard titration as a proxy to obtain pg of citrate synthase. Therefore, the data is presented as pg CS/µg protein. **B**) The same data as depicted in panel (A) is shown but displayed for each cell line individually. Data are based on triplicates, with error bars representing standard deviation (SD).

***FAHD1 oxaloacetate decarboxylation activity with and without additional GDP:***

An enzymatic assay is a fundamental technique used in biochemistry to measure the activity of enzymes. The assay is designed to monitor the decay or conversion of the substrate over time as it interacts with the enzyme. Initially, the substrate is mixed with the enzyme under controlled conditions. As the enzyme catalyzes the reaction, the substrate is converted into one or more products. By measuring the decrease in substrate concentration over time, the rate of the enzymatic reaction can be determined. We employed our developed assay to monitor ODx activity of FAHD1^14^.

After a certain duration, additional molecules were introduced directly into the reaction mix at various concentrations, and the reaction was observed over another period. Numerous substances were screened in a comprehensive endeavor. Among them, only NTPs and DTPs exhibited activity, with GDP demonstrating the most pronounced effect. These findings suggest that GDP might act as a potential activator of FAHD1 activity, aligning well with our model of how FAHD1 contributes to the PEPO-PYR-OAA node. Indeed, considering the oxaloacetate isomerase activity attributed to FAHD1, the observed effects align logically. Thus, the correlation between GDP and FAHD1 activity becomes even more plausible, as it suggests a potential regulatory role of FAHD1 in metabolic processes.

However, control experiments revealed similar effects when oxaloacetate and GDP were combined without FAHD1 presence. Although the relative amplification of the reaction rate was lower in these control experiments compared to those with FAHD1, we consider this data too preliminary for inclusion in the main text and have therefore relegated it to supplementary material. This insight underscores the significance of further investigation into the interplay between FAHD1 and metabolic pathways, shedding light on its broader functional implications in cellular physiology.

**Supplementary Figure S11: Mitochondrial GDP (mGDP) may be a cofactor of FAHD1.** Preliminary experiments suggest a potential augmentation in the catalytic activity of FAHD1 in the presence of mitochondrial GDP. This finding indicates a possible regulatory role of GDP in modulating FAHD1 activity, underscoring its significance in mitochondrial metabolism. Further investigation is warranted to elucidate the mechanistic details and physiological relevance of this interaction, potentially uncovering novel insights into cellular metabolic regulation.

# S8) Mitochondrial content

Mitochondrial content estimation is a crucial aspect of metabolic research, providing insights into cellular energy metabolism and mitochondrial function. Mitochondria are organelles responsible for producing adenosine triphosphate (ATP), the primary energy currency of the cell, through oxidative phosphorylation. Quantifying mitochondrial content allows researchers to assess the abundance of mitochondria within cells or tissues, which is essential for understanding cellular metabolism, energy production, and overall cellular health.

Normalizing metabolic rates to mitochondrial content is important because it helps to account for variations in mitochondrial density among different samples or conditions. By standardizing metabolic rates to mitochondrial content, researchers can accurately compare energy metabolism across different experimental settings, ensuring that observed differences are not solely due to variations in mitochondrial abundance. Moreover, normalizing metabolic rates to mitochondrial content enables researchers to evaluate mitochondrial efficiency and function more accurately. It provides a basis for assessing the relationship between mitochondrial content and metabolic activity, shedding light on how changes in mitochondrial abundance may impact cellular energy metabolism and physiological processes.

For experiments with live-cell imaging, 7x10^4^ cells per compartment were seeded into a four-compartment culture dish (*Greiner*, 627871) a day prior to the experiment. On the day of the experiment cells were stained for mitochondria using *MitoTracker*-Green FM (MTG) (*Invitrogen*, M7514, 200 nM) and Hoechst 33342 (*Thermo-Fisher*, H3570, 1:2000) in PBS. The medium was removed and 500 µL of the staining solution was added. Dishes were incubated for 15 minutes at 37 °C. Afterward, the staining solution was removed, cells were washed two times with PBS and fresh full medium was added. Pictures were taken using the confocal live cell microscope (*Imsol*, CV1000). Mitochondrial content was determined by measuring the CTCF values of each cell individually using the ImageJ software.

**Original data files and statistical evaluations** are provided on reasonable request.

# S9) RT-qPCR quantification

Quantitative Reverse Transcription Polymerase Chain Reaction (RT-qPCR) was employed for quantification purposes (**Fig. S5**). Four U2OS cell lines (hFAHD1.1 O.E, hFAHD1 T192S, hFAHD1 K123A, and control cells) were cultured until reaching 80-90% confluence. After trypsinization, cell counts were conducted. RNA extraction was carried out using the *RNAeasy Kit* (*Qiagen*) with *RTL buffer* and β-Mercaptoethanol. The eluted RNA was quantified using Nanodrop and prepared for cDNA synthesis. For cDNA synthesis, 1 ng of RNA was employed with a cDNA Synthesis kit (*ThermoFisher*, K1622). RT-qPCR was performed with 100 ng of cDNA on a QuantStudioTM 7 Flex System, utilizing the ΔΔCt-method for analysis^15^. The Mastermix was prepared by adding 2 µL of 10x RT Buffer, 0.8 µL of 25x dNTP Mix, 2 µL of 10x RT Random Primers, 1 µL of Reverse Transcriptase, 1 µL of RNAse Inhibitors, and 3.2 µL of Nuclease free H_2_O per reaction, resulting in a total volume of 70 µL for 35 reactions. Primer sequences were designed *via* the NIH Primer-BLAST tool as follows: hFAHD1 (Fwd: 5’-*TGT-GAG-GGT-CAG-GGA-TCT-TC*-3’; Rev: 5’-*GGA-GGA-GTC-CAA-GAA-GAA-GG*-3’), hAKT1 (Fwd: 5’-*GGA-CAA-GGA-CGG-GCA-CAT-TA*-3', Rev: 5’-*CGA-CCG-CAC-ATC-ATC-TCG-TA*-3'), hPCK2 (PEPCK-M) (Fwd: 5’-*GCC-ATC-ATG-CCG-TAG-CAT-C*-3', Rev: 5’*-AGC-CTC-ACT-TCC-ATC-ACA-GAT*-3'), hPC (Fwd: 5’-*ACA-GAG-GTG-AGA-TTG-CCA-TCC*-3', Rev: 5’-*CAC-TGG-ATC-TAC-GTT-GTT-CTC-C*-3'), hSDH (Fwd: 5’-*CTT-GTC-ACC-GAG-CCA-CCA-TT*-3', Rev: 5’-*AAC-ACT-GAC-AAC-CCT-CTC-GC*-3'), hGLS (Fwd: 5’-*GAA-ATT-CGG-AAC-AAG-ACT-GTG*-3', Rev: 5’-*AAC-TTC-GAT-GTG-TCC-TTC-AG*-3'), hGAPDH (Fwd: 5’-*GAT-CTC-GCT-CCT-GGA-AGA-TG*-3', Rev: 5’-*GAG-TCA-ACG-GAT-TTG-GTC-GT*-3')

**Supplementary Figure S5: RT-qPCR gene expression upon FAHD1 OE.** Quantitative Reverse Transcription Polymerase Chain Reaction (RT-qPCR) was utilized for quantification purposes. The expression levels of human genes *Fahd1*, *Akt1*, *Pck2*, *Pc*, *Sdh*, *Gls*, and *Gapdh* were monitored. These findings correspond well with the Western Blot data and support our overarching hypothesis regarding how the metabolic profile of U2OS cells is modulated upon FAHD1 overexpression.

# S10) Scratch assay

The scratch assay, also known as the wound healing assay, is a widely used experimental technique in cell biology to study cell migration and wound repair processes in vitro. This assay mimics the physiological process of wound healing by creating a gap or "scratch" in a confluent monolayer of cells, typically using a sterile pipette tip or a specialized tool. Subsequently, the closure of the scratch is monitored over time, allowing researchers to assess the migratory capacity of cells and the dynamics of wound closure. The scratch assay offers several advantages, including simplicity, cost-effectiveness, and compatibility with a wide range of cell types. It provides a straightforward method to investigate the effects of various factors, such as growth factors, cytokines, drugs, or genetic manipulations, on cell migration and wound healing. Additionally, the scratch assay can be easily combined with live-cell imaging techniques to track individual cell movements in real-time, providing detailed insights into the mechanisms underlying cell migration and wound closure. The migration ability of U2OS was also tested using a defined scratch assay (“wound healing assay”) overnight (**Fig. S6**). The migration of U2OS cells that overexpress the FAHD1 T192S variant was comparable to control cells, while U2OS cells that overexpress the FAHD1 K123A variant migrated slightly slower (**Fig. S7**).

**Supplementary Figure S6: wound healing / scratch assays**. Cells were initially seeded in small dishes, and a gap was created by gently scraping with a 200 µL tip. The migration of cells to close the gap was monitored by capturing images every two hours for a total duration of 24 hours. Images were taken at seven positions along the scratch using the confocal live cell microscope, Cell Voyager CV1000 from Imsol. A: Representative images were captured at the beginning (0 hours), middle (8 hours), and end (20 hours) of each cell line, illustrating the progression of wound closure over time. Black lines delineate the borders of the gap between confluent cells. B: Graphical representations depict the closure of the wound over time, with all cell lines compared to each other. Data comprise curves derived from 3 biological replicates, presented as means ± SEM, with images taken at seven different positions within the scratch for each replicate. GraphPad Prism 5 was utilized for graph generation and statistical analysis.

**Supplementary Figure S7: cell migration assays**. The figures depict migrating cells observed over a 6-hour period, progressing from left to right. Each image corresponds to a specific time point, followed by subsequent time points at 2-hour intervals. Within each image, at least one cell from each cell line is depicted undergoing migration. Movement of individual cells from one position to another during the experiment is indicated by black arrows. These images were captured using the confocal live cell microscope, Cell Voyager CV1000 from Imsol. The graph was generated using the *ScientiFig* plug-in of *ImageJ*.

# S11) Seahorse and Oxygraph data

Seahorse and Oxygraph methodologies are commonly employed in cellular metabolism research to assess cellular respiration and energy production. The Seahorse assay measures cellular respiration rates in real-time by detecting changes in oxygen consumption and extracellular acidification rates. This technique typically involves the sequential injection of compounds that modulate mitochondrial function, allowing for the determination of key parameters such as basal respiration, ATP production, and mitochondrial capacity. On the other hand, Oxygraph assays involve the direct measurement of oxygen consumption rates using a Clark electrode or similar device. Cells or isolated mitochondria are typically incubated in a closed chamber while oxygen concentrations are monitored over time, providing insights into respiratory chain activity and mitochondrial function. Both Seahorse and Oxygraph methodologies offer valuable tools for studying cellular metabolism and are often utilized in conjunction with other biochemical and molecular techniques to elucidate metabolic pathways and mechanisms.

In our experimentation with U2OS cells, we encountered difficulties in uncoupling mitochondrial respiration. Our findings revealed that olygomycin, a compound known for inhibiting complex V of the electron transport chain, effectively influenced cellular respiration in our experimental setup. However, FCCP (carbonyl cyanide-4-(trifluoromethoxy)phenylhydrazone), a commonly used uncoupler of oxidative phosphorylation, did not yield the expected resultson **(Fig. S12a)**. In our high-resolution respirometry experiments using the Oxygraph system, we observed a transient and reproducible induction of respiration upon addition of glutamine (GLN) **(Fig. S12b, S12c, S12d)**. Notably, this effect appeared to be nearly significantly increased in the T192S variant, as evidenced by our initial analysis involving a limited sample size (n=2). We acknowledge that further replicates are necessary to firmly establish the significance of these findings. Despite their preliminary nature, we believe these data are noteworthy as they align with our hypothesis suggesting that the T192S variant may influence U2OS cell metabolism towards glutaminolysis. Therefore, although these findings are presented here in the supplementary material rather than in the main text, they contribute valuable insights to our overall understanding of cellular metabolism in the context of the T192S variant.

**Supplementary Figure S12: high resolution respirometry.** **A**) Attempts to uncouple U2OS cells with FCCP failed in our hands. However, inhibition of complex V was achieved by the addition of oligomycin (OMY). Similar results were observed using high-resolution respirometry (Oxygraph) under different media conditions (data not shown). **B, C, D**) Although we were unable to uncouple U2OS cells in high-resolution respirometry, we observed an interesting and reproducible (albeit temporary) increase in respiration upon adding glutamine (GLN). This effect was nearly significant and was further enhanced in the T192S variant (n=2). Additional replicates are needed to establish significance conclusively. These data align well with our hypothesis that the T192S variant shifts U2OS cell metabolism towards glutaminolysis.

# S12) Vectormaps

The vector maps utilized in this study, along with the *PiggyBac* system used for transfection, are described in the "Materials and Methods" section of the main manuscript. All maps are provided in the Supplementary Material.

The *PiggyBac* system is a versatile tool widely used in molecular biology for efficient transgene delivery and genome manipulation in various cell types and organisms. This system is based on a transposon originally discovered in insects, which can integrate into the host genome with high efficiency and precision. The *PiggyBac* transposase enzyme catalyzes the excision and integration of the transposon, allowing for stable genomic integration of exogenous DNA sequences. Due to its ability to accommodate large DNA inserts and its low genotoxicity, the *PiggyBac* system has become a popular choice for applications such as gene therapy, functional genomics, and transgenic animal generation. Its flexibility, efficiency, and safety profile make it a valuable tool for a wide range of research purposes.

# S13) Western Blot (WB) quantification

The supplementary material accompanying this study includes comprehensive Western Blot data and analysis, meticulously presented alongside statistical findings to ensure transparency and rigor in our research methodology. Furthermore, uncropped Blot Images are provided, offering a complete view of the experimental results. These supplementary resources aim to enhance the reproducibility and clarity of our findings, fostering an environment of scientific integrity and trustworthiness in our work.

While we are currently transitioning to an electronic Chemidoc system, our longstanding practice involved the use of X-ray film for western blot detection, where membrane boundaries remain indiscernible. We provide scans of all X-ray films used as the basis for our analysis. We routinely section our membranes into small pieces to isolate individual protein bands. The "heavily cropped" images found in the main text delineate the borders of these fragments, accompanied by color-line indicators pinpointing the locations of marker bands. It's pertinent to note that original membranes were discarded post-Western blot, given the labor-intensive nature of retaining these fragments. As previously mentioned, full-length scans of the X-ray films are provided below.

**n = 1**

**n = 2**

**n = 3**

**n = 4**

**additional A**

**additional B**

**additional C**

**Uncropped scans of X-ray films for Western Blot detection**

# References:

1.	Mund, A., Coscia, F., Kriston, A., Hollandi, R., Kovács, F., Brunner, A.-D., *et al.* Deep Visual Proteomics defines single-cell identity and heterogeneity. *Nature Biotechnology* **40,** 1231–1240 (2022).

2.	Mund, A., Coscia, F., Kriston, A., Hollandi, R., Kovács, F., Brunner, A.-D., *et al.* Deep Visual Proteomics defines single-cell identity and heterogeneity. *Nature Biotechnology* **40,** 1231–1240 (2022).

3.	Zonta, F., Borgo, C., Quezada Meza, C. P., Masgras, I., Rasola, A., Salvi, M., *et al.* Contribution of the CK2 Catalytic Isoforms α and α’ to the Glycolytic Phenotype of Tumor Cells. *Cells* **10,** (2021).

4.	Guzmán, C., Bagga, M., Kaur, A., Westermarck, J. & Abankwa, D. ColonyArea: An ImageJ Plugin to Automatically Quantify Colony Formation in Clonogenic Assays. *PLoS ONE* **9,** e92444 (2014).

5.	Murphy, M. P., Bayir, H., Belousov, V., Chang, C. J., Davies, K. J. A., Davies, M. J., *et al.* Guidelines for measuring reactive oxygen species and oxidative damage in cells and in vivo. *Nature Metabolism* **4,** 651–662 (2022).

6.	Trott, O. & Olson, A. J. AutoDock Vina: improving the speed and accuracy of docking with a new scoring function, efficient optimization, and multithreading. *Journal of computational chemistry* **31,** 455–61 (2010).

7.	Weiss, A. K. H. H., Loeffler, J. R., Liedl, K. R., Gstach, H. & Jansen-Dürr, P. The fumarylacetoacetate hydrolase (FAH) superfamily of enzymes: multifunctional enzymes from microbes to mitochondria. *Biochemical Society Transactions* **46,** 295–309 (2018).

8.	Hong, H., Seo, H., Park, W. & Kim, K. K.-J. Sequence, structure and function-based classification of the broadly conserved FAH superfamily reveals two distinct fumarylpyruvate hydrolase subfamilies. *Environmental microbiology* **22,** 270–285 (2020).

9.	Krömer, J. O., Sorgenfrei, O., Klopprogge, K., Heinzle, E. & Wittmann, C. In-depth profiling of lysine-producing Corynebacterium glutamicum by combined analysis of the transcriptome, metabolome, and fluxome. *Journal of bacteriology* **186,** 1769–84 (2004).

10.	Ran, T., Wang, Y., Xu, D. & Wang, W. Expression, purification, crystallization and preliminary crystallographic analysis of Cg1458: A novel oxaloacetate decarboxylase from Corynebacterium glutamicum. *Acta Crystallographica Section F: Structural Biology and Crystallization Communications* **67,** 968–970 (2011).

11.	Ran, T., Gao, Y., Marsh, M., Zhu, W., Wang, M., Mao, X., *et al.* Crystal structures of Cg1458 reveal a catalytic lid domain and a common catalytic mechanism for the {FAH} family. *Biochemical Journal* **449,** 51–60 (2012).

12.	Weiss, A. K. H., Naschberger, A., Loeffler, J. R., Gstach, H., Bowler, M. W., Holzknecht, M., *et al.* Structural basis for the bi-functionality of human oxaloacetate decarboxylase FAHD1. *Biochemical Journal* **475,** 3561–3576 (2018).

13.	Weiss, A. K. H., Holzknecht, M., Cappuccio, E., Dorigatti, I., Kreidl, K., Naschberger, A., *et al.* Expression, Purification, Crystallization, and Enzyme Assays of Fumarylacetoacetate Hydrolase Domain-Containing Proteins. *Journal of Visualized Experiments* e59729 (2019). doi:10.3791/59729

14.	Weiss, A. K. H., Naschberger, A., Loeffler, J. R., Gstach, H., Bowler, M. W., Holzknecht, M., *et al.* Structural basis for the bi-functionality of human oxaloacetate decarboxylase FAHD1. *Biochemical Journal* **475,** 3561–3576 (2018).

15.	Rao, X., Huang, X., Zhou, Z. & Lin, X. An improvement of the 2ˆ(-delta delta CT) method for quantitative real-time polymerase chain reaction data analysis. *Biostatistics, bioinformatics and biomathematics* **3,** 71–85 (2013).
